# Effectiveness of physical activity interventions delivered or prompted by health professionals in primary care settings: systematic review and meta-analysis of randomised controlled trials

**DOI:** 10.1136/bmj-2021-068465

**Published:** 2022-02-23

**Authors:** Victoria E Kettle, Claire D Madigan, April Coombe, Henrietta Graham, Jonah J C Thomas, Anna E Chalkley, Amanda J Daley

**Affiliations:** 1 The Centre for Lifestyle Medicine and Behaviour (CLiMB), The School of Sport, Exercise and Health Sciences, Loughborough University, Loughborough, UK; 2 Institute of Applied Health Research, University of Birmingham, Birmingham, UK

## Abstract

**Objective:**

To examine the effectiveness of physical activity interventions delivered or prompted by primary care health professionals for increasing moderate to vigorous intensity physical activity (MVPA) in adult patients.

**Design:**

Systematic review and meta-analysis of randomised controlled trials.

**Data sources:**

Databases (Medline and Medline in progress, Embase, PsycINFO, CINAHL, SPORTDiscus, Sports Medicine and Education Index, ASSIA, PEDro, Bibliomap, Science Citation Index, Conference Proceedings Citation Index), trial registries (Cochrane Central Register of Controlled Trials, ClinicalTrials.gov, TRoPHI), and grey literature (OpenGrey) sources were searched (from inception to September 2020).

**Eligibility criteria for selecting studies:**

Randomised controlled trials of aerobic based physical activity interventions delivered or prompted by health professionals in primary care with a usual care control group or another control group that did not involve physical activity.

**Study selection and analysis:**

Two independent reviewers screened the search results, extracted data from eligible trials and assessed the risk of bias using the Cochrane risk of bias tool (version 2). Inverse variance meta-analyses using random effects models examined the primary outcome of difference between the groups in MVPA (min/week) from baseline to final follow-up. The odds of meeting the guidelines for MVPA at follow-up were also analysed.

**Results:**

14 566 unique reports were identified and 46 randomised controlled trials with a range of follow-ups (3-60 months) were included in the meta-analysis (n=16 198 participants). Physical activity interventions delivered or prompted by health professionals in primary care increased MVPA by 14 min/week (95% confidence interval 4.2 to 24.6, P=0.006). Heterogeneity was substantial (I^2^=91%, P<0.001). Limiting analyses to trials that used a device to measure physical activity showed no significant group difference in MVPA (mean difference 4.1 min/week, 95% confidence interval −1.7 to 9.9, P=0.17; I^2^=56%, P=0.008). Trials that used self-report measures showed that intervention participants achieved 24 min/week more MVPA than controls (95% confidence interval 6.3 to 41.8, P=0.008; I^2^=72%, P<0.001). Additionally, interventions increased the odds of patients meeting guidelines for MVPA by 33% (95% confidence interval 1.17 to 1.50, P<0.001; I^2^=25%, P=0.11) versus controls. 14 of 46 studies were at high risk of bias but sensitivity analyses excluding these studies did not alter the results.

**Conclusions:**

Physical activity interventions delivered or prompted by health professionals in primary care appear effective at increasing participation in self-reported MVPA. Such interventions should be considered for routine implementation to increase levels of physical activity and improve health outcomes in the population.

**Systematic review registration:**

PROSPERO CRD42021209484.

## Introduction

Physical inactivity is a leading global risk factor for mortality and morbidity.^1^ The World Health Organization updated their physical activity guidelines in 2020 and now state that adults should undertake at least 150-300 minutes of moderate intensity physical activity, or 75-150 minutes of vigorous intensity physical activity, or an equivalent combination of aerobic based physical activity each week.^2^ Current national physical activity programmes have been ineffective in most countries,^3^ with one in four adults insufficiently physically active and no improvement in participation rates evident over the past two decades.^4^ The World Health Assembly has set a target to reduce physical inactivity by 15% by 2030.^5^ This target includes a recommendation for all countries to integrate physical activity counselling programmes into primary healthcare. On average, 70-80% of adults visit their general practice at least once each year.^6^ Therefore, health professionals in primary care have a unique opportunity to routinely prompt and provide physical activity interventions to patients through the millions of health consultations that take place worldwide each week.

Previous reviews have investigated the effectiveness of physical activity interventions delivered in primary care settings and some of these have reported small to moderate effects depending on the inclusion criteria used.^7^ ^8^ ^9^ ^10^ ^11^ ^12^ ^13^ ^14^ ^15^ However, these reviews have not been able to offer definitive conclusions to guide implementation or health policy on this question for several reasons: they were narrative reports,^7^ ^11^ ^12^ included non-randomised trials,^7^ ^8^ ^10^ ^12^ recruited specific clinical populations,^11^ ^13^ the findings were based solely on self-report measures of physical activity,^8^ ^10^ ^11^ ^13^ ^15^ or they included interventions not delivered or prompted by health professionals in primary care.^7^ ^9^ ^11^ ^14^ More recent reviews have only investigated outcomes such as energy expenditure^13^ and total amounts of physical activity,^14^ ^15^ making it unclear how effective primary care delivered physical activity interventions are for increasing moderate to vigorous intensity physical activity (MVPA), the required intensity to meet WHO physical activity guidelines.^2^ This systematic review and meta-analysis aimed to robustly and comprehensively synthesise evidence from randomised controlled trials on whether physical activity interventions delivered or prompted by health professionals in primary care are effective in increasing MVPA in their patients.

## Methods

This systematic review and meta-analysis has been reported according to the preferred reporting items for systematic reviews and meta-analyses (PRISMA).^16^ The protocol was registered with the international prospective register of systematic reviews (PROSPERO) on 1 February 2021 (CRD42021209484).

### Eligibility criteria

Randomised controlled trials were eligible when adult participants or clusters were randomly allocated to a physical activity intervention or a usual care control group, or another control group that did not involve physical activity. No other restrictions were applied relating to personal characteristics. Any type of predominately aerobic based physical activity intervention delivered or prompted (referred to here as delivered) by a health professional in a primary care setting was eligible. Prompted refers to interventions where the primary care health professional was involved in the intervention but an additional interventionalist (eg, a physical activity counsellor) was also involved in intervention delivery. Delivered refers to interventions delivered solely by primary care health professionals. Interventions aimed entirely at body conditioning (eg, yoga, tai-chi) were excluded because these types of activities do not involve an aerobic component and are unlikely to increase levels of MVPA. All primary care settings were included, broadly defined as the first point of contact in the healthcare system providing accessible, continued, comprehensive, and coordinated care, which focuses on people’s long term health rather than short disease durations.^17^ Trials were included when at least one interaction took place between health professionals in primary care and patients.

We excluded trials evaluating exercise referral schemes because primary care health professionals would only be acting as referral mechanisms rather than being directly involved in delivering interventions. Rehabilitation trials were excluded because patients might have limited capability to perform MVPA. Trials that assessed interventions lasting four weeks or longer were eligible and were required to have at least one follow-up beyond baseline.

Trials were required to report data (in continuous or dichotomous units) related to participation in MVPA from baseline to final follow-up or provide data that allowed this to be calculated. Studies measuring MVPA at follow-up but not at baseline were also eligible in line with the Cochrane handbook.^18^ No restrictions were made on the method used to assess MVPA, with data from self-report and device measures included, or on publication type, year, or language.

### Search strategy

The search strategy was devised and tested in Medline, combining intervention and setting terms with established randomised controlled trial filters. We adapted the search for the following datasets: Embase, PsycINFO, CINAHL, SPORTDiscus, Sports Medicine and Education Index, ASSIA, PEDro, Bibliomap, Science Citation Index (SCI-E), Conference Proceedings Citation Index (CPCI-S), and OpenGrey. We searched the following trial registers: Cochrane Central Register of Controlled Trials, ClinicalTrials.gov, and TRoPHI. The supplementary material provides the full search strategy. No date limitations were applied except for SCI-E, where dates were restricted to the past 10 years for manageability. AC performed the searches between 9 and 21 September 2020. Subsequently, a brief search of PubMed covering the six months after these search dates was performed before the final analyses (1 April 2021).

### Study selection and data extraction

Duplicates were removed automatically in EndNote version X9 (Clarivate, Philadelphia, Pennsylvania) and the remaining results were uploaded to Covidence systematic review software,^19^ where additional duplicates were removed. Two independent reviewers from VEK, AJD, CDM, HG, and JJCT screened study titles and abstracts, applying the eligibility criteria except for SCI-E, CPCI-S, TRoPHI, Bibliomap, ClinicalTrials.gov, and Open Grey results which were single screened (VEK). The full texts of potentially eligible studies were retrieved and assessed independently by VEK and AJD or AEC. All decisions of inclusion or exclusion were automatically recorded in Covidence, and reviewers were blinded to each other’s decisions. Any disagreements were discussed between the two reviewers and resolved by consensus. All included full texts were examined and multiple reports from the same trial were merged in Covidence before data extraction.

Data were extracted about the characteristics of included studies and summarised (table 1, table 2). All outcome data used for the meta-analysis were independently extracted by VEK and HG or AEC. Disagreements were discussed, and the original paper was consulted to reach consensus. Corresponding authors were contacted by email if data were unreported or additional details were required.

**Table 1:** Characteristics of included studies, according to study author surname (beginning with A-H)

Study (year), country	RCT type	Participants	Intervention	Comparisons*	Follow-up (months)	MVPA measure
Aittasalo (2006),^20^ Finland	Cluster	n=265, 24% male, 20-65 years; general population, inactive	PA, brief, GP	Usual care	6	Self-report: IPAQ
Alonso-Dominguez (2019),^21^ Spain	Individual	n=204, 54% male, 25-70 years; type 2 diabetes mellitus, PA not part of eligibility	PA and diet, intensive, nurse	Usual care	12	Self-report: IPAQ
Apinaniz (2019),^22^ Spain	Individual	n=110, 28% male, 18-45 years; body mass index ≥25, inactive	PA and diet, brief, nurse or GP	Usual care	6	Self-report: degree of adherence to recommendations
Arija (2018),^23^ Spain	Individual	n=207, 23% male, ≥18 years; people with hypertension, PA not part of eligibility	PA, intensive, nurse	Usual care	9	Self-report: IPAQ
Carroll (2010),^24^ US	Cluster	n=394, 31% male, adults, mean age 46.4 years†; general population, inactive	PA, brief, GP	General preventive screening report	6	Self-report: 7d-PAR
Cheng (2018),^25^ US	Individual	n=404, 60% male, ≥40 years; survivors of ischaemic stroke or transient ischaemic attack, PA not part of eligibility	Stroke prevention including PA, intensive, nurse or physician assistant	Usual care	12	Self-report: MVPA ≥3 days/week
Clapperton (2020),^26^ Trinidad	Individual	n=130, 14% male, ≥18 years; general population, inactive	PA, multiple brief, GP	Usual care	10	Self-report: Brief assessment tool
Driehuis (2012),^27^ Netherlands	Individual	n=457, 48% male, 40-70 years; body mass index 25-40 and hypertension or dyslipidemia, PA not part of eligibility	PA and diet, intensive, nurse	Usual care	36	Self-report: SQUASH
Dubbert (2008),^28^ US	Individual	n=224, 100% male, 60-85 years; veterans with physical function limitations, inactive	PA, multiple brief, nurse	Nurse health discussion	10	Device measured: RT3 triaxial accelerometer
Duijzer (2017),^29^ Netherlands	Individual	n=316, 52% male, 40-70 years; increased risk of type 2 diabetes, PA not part of eligibility	PA and diet, intensive, physiotherapist	Usual care	18	Self-report: SQUASH
Dutton (2006),^30^ US	Cluster	n=139, 0% male, 18-65 years; low-income African American women BMI ≥25, PA not part of eligibility.	PA, multiple brief, GP	Usual care	6	Self-report: 7d-PAR
Elley (2003),^31^ New Zealand	Cluster	n=878, 34% male, 40-79 years; general population, inactive	PA, multiple brief, primary care health professional and exercise specialist	Usual care	12	Self-report: Auckland heart study questionnaire
Fortier (2011),^32^ Canada	Individual	n=120, 31% male, 18-69 years; general population, inactive	PA, intensive, GP and PA counsellor	Brief GP counselling	6	Device measure: Actical
Garcia-Ortiz (2018),^33^ Spain	Individual	n=833, 38% male, <70 years; general population, PA not part of eligibility	PA and diet, multiple brief, nurse	Brief nurse counselling	12	Device measure: ActiGraph
Goldstein (1999),^34^ US	Cluster	n=355, 76% male, ≥50 years; general population, inactive	PA, multiple brief, GP and researcher	Usual care	8	Self-report: PASE
Gomez-Huelgas (2015),^35^ Spain	Individual	n=601, 55% male, 18-80 years; metabolic syndrome patients, PA not part of eligibility	PA and diet, multiple brief, nurse	Usual care	36	Self-report: Minnesota Leisure-Time PA Questionnaire
Grandes (2011),^36^ Spain	Cluster	n=4317, 35% male, 20-80 years; general population, inactive	PA, brief, GP	Usual care	24	Self-report: 7d-PAR
Hall (2011),^37^ Morey (2009),^38^ US	Individual	n=234, 100% male, ≥70 years; older adults with multiple morbidities, inactive	PA, multiple brief, GP and lifestyle counsellor	Usual care	24‡	Self-report: CHAMPS
Harari (2008),^39^ UK	Cluster	n=2503, 46% male, >65 years; general population, PA not part of eligibility	Lifestyle including PA, brief, GP	Usual care	12	Self-report: PASE
Hardeman (2020),^40^ UK	Individual	n=1007, 38% male, 40-74 years; general population, PA not part of eligibility	PA, brief, primary care practitioner	Usual care	3	Device measure: ActiGraph
Harris (2012),^41^ Australia	Cluster	n=699, 43% male, 40-64 years; hypertension, hyperlipidaemia or aged 56-64 years, PA not part of eligibility	PA, diet and lifestyle, intensive, GP or nurse and dietician or exercise specialist	Usual care	12	Self-report: Brief assessment tool
Harris (2018 PACE-Lift),^42^ UK	Cluster	n=298, 46% male, 60-75 years; general population, PA not part of eligibility	PA, intensive, nurse	Usual care	48	Device measure: ActiGraph
Harris (2018 PACE-UP),^42^ UK	Cluster	n=1023, 36% male, 45-75 years; general population, inactive	PA, multiple brief, nurse	Usual care	36	Device measure: ActiGraph
Hellgren (2020),^43^ Sweden	Individual	n=123, 42% male, 35-75 years; individuals with prediabetes, PA not part of eligibility	PA and lifestyle, intensive, nurse	Usual care	60	Self-report: leisure time PA per week
Hesselink (2013),^44^ Netherlands	Cluster	n=366, 53% male, ≥45 years; individuals with impaired fasting glucose, PA not part of eligibility	PA and diet, multiple brief, nurse	Usual care	24	Self-report: SQUASH
Huebschmann (2018),^45^ US	Individual	n=50, 50% male, 50-85 years; patients with type 2 diabetes, inactive	PA, multiple brief, GP and clinic staff coach	Enhanced usual care: printed materials and mailings	3	Device measure: ActiGraph

The term GP includes physician, clinician, doctor, general practitioner.

7d-PAR=7-day Physical Activity Recall; CHAMPS=Community Healthy Activities Model Program for Seniors; IPAQ=International Physical Activity Questionnaire (short form); MVPA=moderate to vigorous intensity physical activity; PA=physical activity; PASE=Physical activity Scale for the Elderly; RCT=randomised controlled trial; SQUASH=Short Questionnaire to assess health-enhancing physical activity.

* Usual care as stated in paper.

† Mean age stated when age range was not reported in study.

‡ Adherence to physical activity guidelines assessed at 12 months only.

**Table 2:** Characteristics of included studies, according to study author surname (beginning with J-Y)

Study (year), country	RCT type	Participants	Intervention	Comparisons*	Follow-up (months)	MVPA measure
Jimmy (2005),^46^ Switzerland	Individual	n=161, 42% male, >15 years; general population, inactive	PA, intensive, GP and PA specialist	GP feedback on current PA levels	14	Self-report: 7-day recall questionnaire
Jolly (2017),^47^ UK	Individual	n=577, 63% male, >18 years; patients with mild COPD, PA not part of eligibility	COPD self-management including PA, intensive, nurse	Usual care	12	Device measure: GENEActive
Kloek (2018),^48^ Netherlands	Cluster	n=204, 32% male, 40-80 years; people with hip or knee osteoarthritis, inactive	PA, multiple brief, physiotherapist	Usual care	12	Device measure: ActiGraph
Lawton (2008),^49^ New Zealand	Individual	n=1089, 0% male, 40-74 years; general population, inactive	PA, multiple brief, nurse and exercise facilitator	Usual care	24	Self-report: NZPAQ-LF
Migneault (2012),^50^ US	Individual	n=337, 30% male, ≥35 years; African Americans with hypertension, PA not part of eligibility	PA, diet and drugs adherence, multiple brief, GP and researcher	Educational session	8	Self-report: 7d-PAR
Mitchell (2013),^51^ UK	Individual	n=184, 55% male, mean age 69 years†; patients with COPD, PA not part of eligibility	COPD self-management including PA, intensive, physiotherapist	Usual care	6	Device measure: Sensewear
Moreno (2019),^52^ Spain	Individual	n=594, 60% male, ≥18 years; patients with type 2 diabetes, PA not part of eligibility	Diabetes self-management including PA, intensive, healthcare professional	Usual care	24	Self-report: 7d-PAR
Morey (2012),^53^ US	Individual	n=302, 97% male, 60-89 years; older adults with prediabetes mellitus, inactive	PA, multiple brief, GP and lifestyle counsellor	Usual care	12	Self-report: CHAMPS
Pears (2016),^54^ UK	Individual	n=394, 41% male, 40-74 years; general population, inactive	PA, brief, nurse or healthcare assistant	Usual care	1	Device measure: ActiGraph
Pinto (2002),^55^ US	Individual	n=298, 28% male, ≥25 years; general population, inactive	PA, brief, GP and researcher	Healthy eating intervention	6	Self-report: 7d-PAR
Pinto (2005),^56^ US	Individual	n=100, 37% male, ≥60 years; older adults, inactive	PA, intensive, GP and health educator	Brief advice only	6	Self-report: 7d-PAR
Reed (2008),^57^ US	Individual	n=237, 27% male, adults; general population, PA not part of eligibility	PA, brief, GP or nurse	Usual care	2	Self-report: IPAQ
Richardson (2007),^58^ US	Individual	n=20, 25% male, ≥60 years; geriatric population, inactive	PA, brief, GP	Usual care	1	Self-report: MVPA hours during past 7 days
Schillinger (2009),^59^ US	Individual	n=339, 41% male, >17 years; patients with type 2 diabetes, PA not part of eligibility	Diabetes self-management including PA, intensive, GP and health educator	Usual care	12	Self-report: mins of MVPA on each of the past 7 days
Steptoe (1999),^60^ UK	Cluster	n=883, 46% male, 18-69 years; adults at increased risk of coronary heart disease, inactivity was one of the possible inclusion criteria	PA, smoking and diet, multiple brief, nurse	Usual care	12	Self-report: Allied Dunbar National Fitness Survey
Taheri (2020),^61^ Qatar	Individual	n=158, 73% male, 18-50 years; patients with early type 2 diabetes, PA not part of eligibility	PA and diet, intensive, GP and dietician or personal trainer	Usual care	12	Self-report: IPAQ
Tiessen (2013),^62^ Netherlands	Individual	n=201, 69% male, 50-75 years; patients with increased cardiovascular risk, inactivity was one of the possible inclusion criteria	CVD risk including PA, multiple brief, nurse	Usual care	12	Self-report: SQUASH
Valve (2013),^63^ Finland	Cluster	n=3059, 0% male, 17-21 years; young women, PA not part of eligibility	PA, diet and sleep, multiple brief, nurse	Sexual health and standard lifestyle counselling	30	Self-report: based on previous Finnish health behaviour questionnaires
Van der Weegan (2015),^64^ Netherlands	Cluster	n=199, 49% male, 40-70 years; patients with COPD or type 2 diabetes, inactive	PA, multiple brief, nurse	Usual care	9	Device measure: Personal activity monitor
Van Sluijs (2005),^65^ Netherlands	Cluster	n=771, 51% male, 18-70 years; patients with hypertension, hypercholesterolemia or non-insulin dependent diabetes, inactive	PA, multiple brief, GP or nurse and PA counsellor	Usual care	12	Self-report: SQUASH
Vermunt (2012),^66^ Netherlands	Individual	n=925, 46% male, 40-70 years; patients at high risk of type 2 diabetes, PA not part of eligibility	PA and diet, intensive, GP or nurse and dietician or physiotherapist.	Usual care	30	Self-report: SQUASH
Volger (2013),^67^ US	Individual	n=390, 20% male, ≥21 years; obese (body mass index 30-50), PA not part of eligibility	PA and diet, multiple brief, GP and medical assistant	Usual care	24	Self-report: Paffenbarger PA survey
Westland (2020),^68^ Netherlands	Cluster	n=195, 61% male, 40-75 years; patients at risk of CVD, inactive	PA, multiple brief, nurse	Usual care	6	Device measure: Personal activity monitor
Writing Group for the Activity Counselling Trial Research Group (2001),^69^ US	Individual	n=874, 55% male, 35-75 years; general population, inactive	PA, intensive, GP and health educator (two groups)	Usual care	24	Self-report: 7d-PAR
Yates (2017),^70^ UK	Cluster	n=808, 64% male, 18-74 years; adults with a high risk of type 2 diabetes, PA not part of eligibility	PA, intensive, GP or health educator	Advice leaflet	36	Device measure: ActiGraph

The term GP includes physician, clinician, doctor, general practitioner.

7d-PAR=7-day Physical Activity Recall; CHAMPS=Community Healthy Activities Model Program for Seniors; COPD=chronic obstructive pulmonary disease; CVD=cardiovascular disease; IPAQ=International Physical Activity Questionnaire (short form); MVPA=moderate-to-vigorous intensity physical activity; NZPAQ-LF=Long form of the New Zealand PA questionnaire; PA=physical activity; RCT=randomised controlled trial; SQUASH=Short Questionnaire to assess health-enhancing physical activity.

* Usual care as stated in paper.

† Mean age stated when age range was not reported in study.

### Risk of bias and quality of evidence assessment

Two independent reviewers (VEK, CDM) assessed the risk of bias using the Cochrane risk of bias tool (version 2, ROB2).^71^ Any disagreements between the reviewers were discussed and resolved through consensus by referring to the full text. Figures for risk of bias were produced using ROB2 and funnel plots were created using RevMan 5.4.1^72^ to assess risk of publication bias.

### Outcomes and data synthesis

The primary outcome was minutes of MVPA each week. The proportion of participants meeting guidelines for MVPA was also included as an important secondary outcome. Other secondary outcomes were total physical activity and sedentary time. We selected these secondary outcomes because strong evidence has shown that any increase in physical activity, regardless of intensity, is also important for health,^73^ with similar outcomes for reducing sedentary behaviours.^74^ Data for weight and body mass index were also synthesised when reported in the included studies because evidence reports that physical activity can be important for weight management.^13^ ^75^

Inverse variance meta-analyses using random effects models were conducted in RevMan using weighted mean differences and 95% confidence intervals to describe between group differences for change in MVPA (min/week). We used random effects models because of the variety of physical activity interventions tested and the likelihood of different intervention effects. For trials with a high loss to follow-up (>20%), change in MVPA was calculated using baseline MVPA observed carried forward^76^ (n=10 trials). We excluded two trials from this analysis because MVPA was measured only at follow-up in one trial^54^ and the other reported medians.^51^

The likelihood of meeting MVPA guidelines (according to the guidance used in the individual randomised controlled trials) was explored using odds ratios and 95% confidence intervals. We calculated the standardised mean difference for total physical activity because of the variety of outcomes reported (eg, minutes, accelerometer counts, steps) and the effect size was interpreted as small (0.2), moderate (0.5), or large (0.8).^77^ See supplementary material for additional information on data synthesis methods.

Prespecified subgroup analyses were conducted that compared device and self-report measures of MVPA. We investigated intervention intensity according to the number of contacts with an interventionalist (at least one of which must have been with a health professional in primary care) to compare the effects of brief (one session ≤30 min),^8^ multiple brief (more than one session ≤30 min), and intensive interventions (more than one session >30 min) on outcomes. A sensitivity analysis was conducted excluding the studies considered at high risk of bias.

Post hoc subgroup analyses were conducted that compared interventions with a high number of intervention contacts (at least five contacts) versus a low number (less than five contacts). We investigated the merits of interventions delivered solely by primary care health professionals versus those involving primary care health professionals plus other interventionalists. Because primary healthcare systems differ by country, the impact of country was examined. We also explored the effect of difference in follow-up length (0-6, 7-12, >12 months).

### Patient and public involvement

No patients or the public were involved in this systematic review due to funding restrictions.

## Results

**Fig 1:**
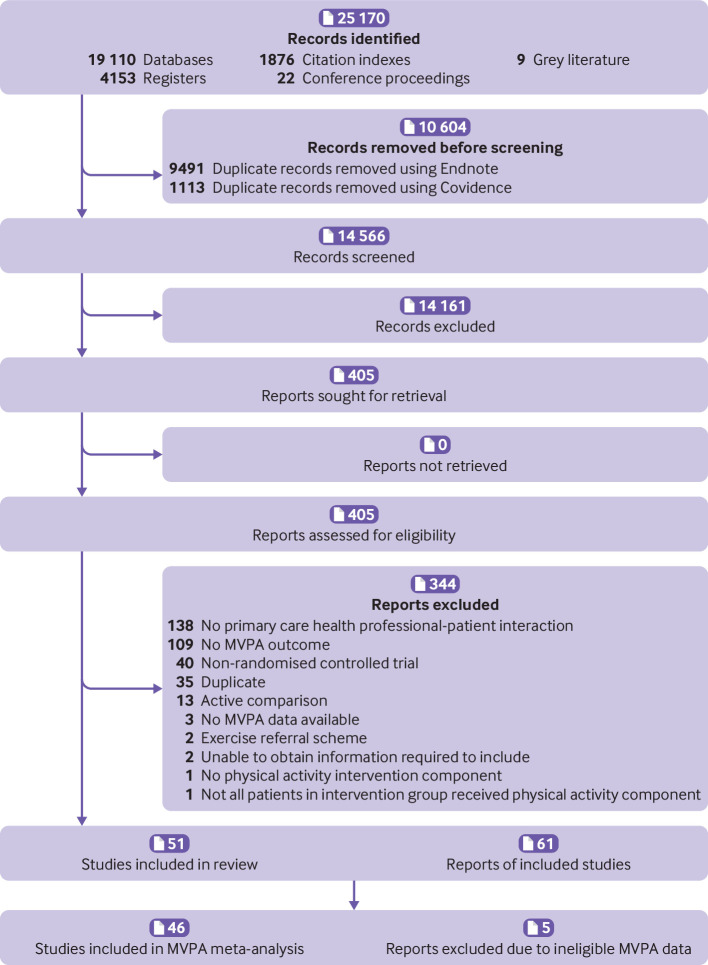
PRISMA (preferred reporting items for systematic reviews and meta-analyses) flow diagram

A total of 25 170 reports were identified from searches; 14 566 titles and abstracts were screened after removing duplicates. Of these, 405 full texts were assessed with 61 reports of 51 studies included in the review; 46 of these were included in the meta-analysis (fig 1). Five studies could not be meta-analysed because MVPA was not reported in a unit that would allow the data to be aggregated (kcal/week,^67^ episodes/week,^60^ MVPA score,^58^ unclear units,^57^ or ineligible for baseline observation carried forward analysis^54^). Of 33 study authors contacted, 10 provided further information^23^ ^25^ ^78^ ^79^ or data.^26^ ^35^ ^41^ ^43^ ^45^ ^65^

Most studies were conducted in the United States (n=16), the UK (n=9), the Netherlands (n=9), and Spain (n=7), with the remainder in Finland, New Zealand, Trinidad, Canada, Australia, Sweden, Switzerland, and Qatar (table 1, table 2). Thirty three studies were individual randomised controlled trials and 18 were cluster randomised controlled trials. Most trials recruited participants at increased risk of disease or diseased populations (n=30) and/or inactive participants (n=24). Physical activity was the primary focus in most interventions (n=33) followed by physical activity and dietary behaviours (n=10), with others focusing on multiple health behaviours that included physical activity (n=8). GPs, nurses, and physiotherapists delivered the interventions in most trials (n=31), with others involving additional interventionalists including health educators or counsellors, exercise specialists, dieticians, and researchers. About half of the interventions were delivered in multiple brief sessions (n=23), 18 were intensive, and 10 were brief. The number of contacts with an interventionalist ranged from 1 to 72; 23 trials involved fewer than five contacts and the remainder five or more contacts (n=28). The control group was generally usual care (n=40). The length of follow-up ranged from one month to five years (see supplementary table 1 for intervention details). MVPA was measured using self-report in most trials (n=37) and using a device in 14 trials.

**Fig 2:**
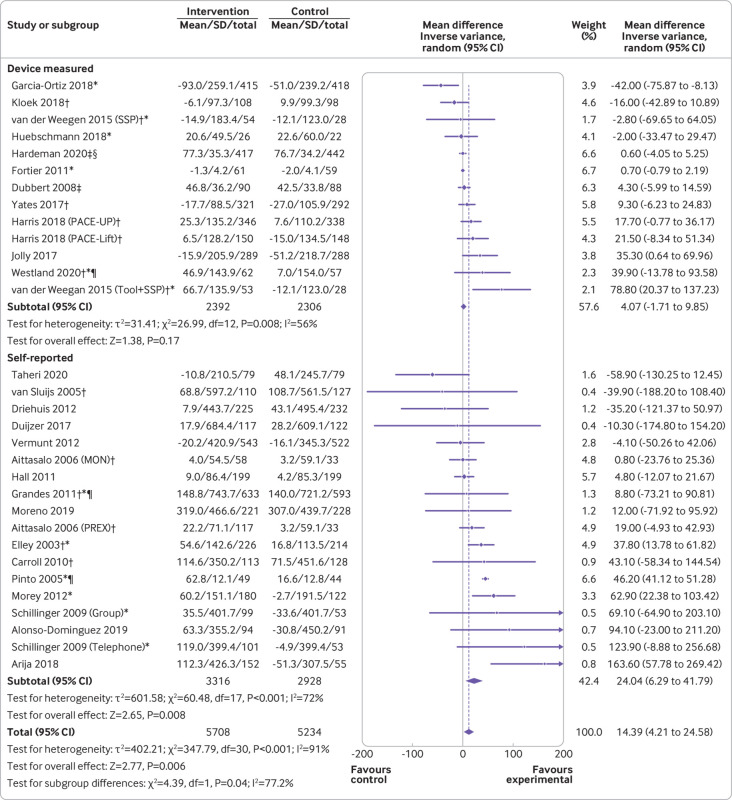
Moderate to vigorous intensity physical activity (MVPA min/week) with measurement type subgroups (device measured or self-reported). *Intention to treat analysis; †cluster randomised controlled trial; ‡follow-up values; §geometric means; ¶adjusted means. SD=standard deviation

Physical activity interventions delivered by health professionals in primary care significantly increased MVPA versus control groups (mean difference 14.4 min/week, 95% confidence interval 4.2 to 24.6, P=0.006; fig 2). Heterogeneity was substantial (I^2^=91%, P<0.001). Limiting analyses to only trials that used a device to measure physical activity showed no significant group difference in MVPA (4.1 min/week, −1.7 to 9.9, P=0.17; I^2^=56%, P=0.008). Trials that used self-report measures showed that intervention participants reported achieving 24 min/week more MVPA than controls (95% confidence interval 6.3 to 41.8, P=0.008; I^2^=72%, P<0.001). No difference was found in minutes per week of MVPA between the groups based on the intensity of the intervention, but interventions with at least five contacts had a larger effect compared with those with less than five contacts for self-reported minutes per week of MVPA (table 3). Furthermore, interventions delivered by primary care health professionals in combination with other interventionalists significantly increased self-reported MVPA, whereas interventions delivered by primary care health professionals alone did not. No subgroup differences were observed for device measured minutes per week of MVPA.

**Table 3:** Results of subgroup analyses stratified by self-reported and device measured moderate to vigorous intensity physical activity (MVPA, min/week)

Analysis and subgroups	No of participants	Mean difference (95% CI)	P value	Heterogeneity, I^2^ (%) (P value)
Self-reported MVPA min/week				
Intervention intensity*				
Brief	1708	11.0 (−5.6 to 27.5)	0.19	0 (0.69)
Multiple brief	1377	28.4 (−2.3 to 59.2)	0.07	70 (0.02)
Intensive	3159	29.6 (−7.0 to 66.2)	0.11	63 (0.004)
Interventionalist				
PCHP only	3245	19.7 (−6.1 to 45.5)	0.13	36 (0.13)
PCHP and other	2999	25.9 (2.8 to 49.1)	0.03	78 (<0.001)
No of interventionalist contacts				
<5	2385	19.0 (4.5 to 33.6)	0.01	6 (0.38)
≥5	3859	28.7 (2.6 to 54.7)	0.03	76 (<0.001)
Device measured MVPA min/week				
Intervention intensity				
Brief	859	0.6 (−4.1 to 5.3)	0.80	NA†
Multiple brief	2231	4.1 (−13.0 to 21.1)	0.64	64 (0.007)
Intensive	1608	9.3 (−3.1 to 21.8)	0.14	56 (0.08)
Interventionalist				
PCHP only	3917	7.3 (−4.2 to 18.7)	0.21	64 (0.003)
PCHP and other	781	0.8 (−0.7 to 2.3)	0.31	0 (0.55)
No of interventionalist contacts				
<5	3533	13.5 (−4.2 to 31.2)	0.13	70 (0.002)
≥5	1165	0.8 (−0.7 to 2.3)	0.29	0 (0.53)

PCHP=primary care health professional.

* Brief (one session ≤30 min), multiple brief (more than one session ≤30 min), intensive (more than one session >30 min).

† Results based on one study.

When MVPA data (self-report and device measured combined) were stratified by country, larger intervention effectiveness was seen in trials conducted in the US and the UK compared with Spain, the Netherlands, and other countries (supplementary fig 1). Follow-up lengths of seven months or longer were effective at increasing minutes per week of MVPA, with the largest effect seen in follow-up lengths of 7-12 months (supplementary fig 2).

Five studies could not be included in the meta-analysis of minutes per week of MVPA; significant increases in MVPA (self-report) were reported by Volger and colleagues^67^ for the brief and enhanced brief lifestyle counselling groups (+593.4±175.9 and +415.4±179.6 kcal/week, respectively) compared with usual care (70.4 ±185.5 kcal/week) at 24 month follow-up. Steptoe and colleagues^60^ found that the intervention group increased the number of episodes of MVPA in the past four weeks (self-report) compared with the control group (+3.9 sessions, 95% confidence interval 1.0 to 6.8) after one year. Conversely, Pears and colleagues^54^ found no difference in device measured MVPA after one month with any of the three brief interventions tested compared with the usual care group. Richardson^58^ showed no difference between the intervention and control groups for MVPA (self-report). The study by Reed and colleagues^57^ was excluded from the MVPA meta-analysis because the units used were unclear and no response was received from the author.

**Fig 3:**
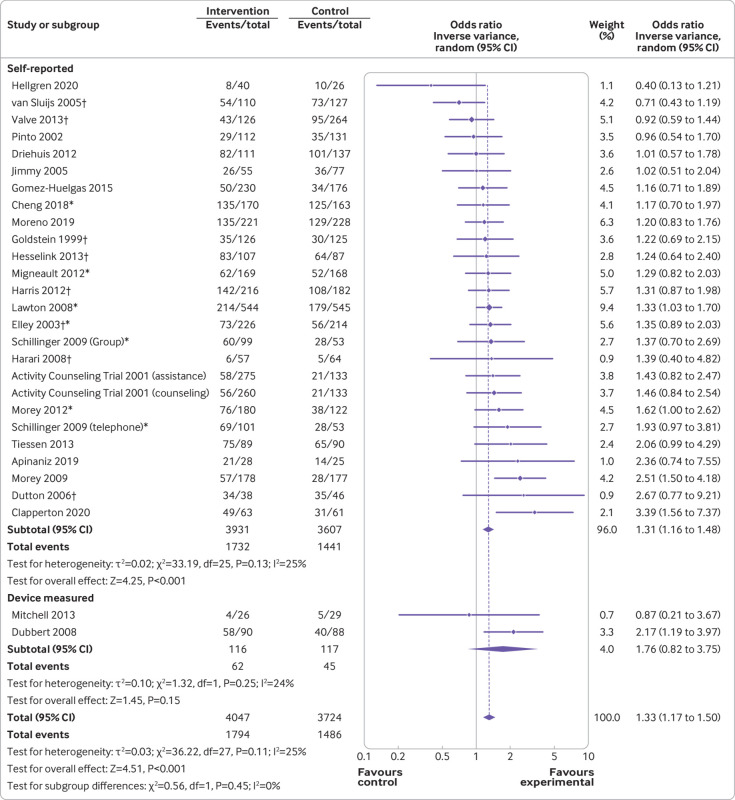
Proportion of participants meeting moderate to vigorous intensity physical activity guidelines with measurement type subgroups. *Intention to treat analysis; †cluster randomised controlled trial

The proportion of participants meeting guidelines for MVPA was significantly higher in the intervention group versus the control group (odds ratio 1.33, 95% confidence interval 1.17 to 1.50, P<0.001), with low heterogeneity (I^2^=25%, P=0.11). In trials that assessed MVPA using self-report measures, this effect remained (1.31, 1.16 to 1.48, P<0.001; I^2^=25%, P=0.13), but not when the analysis was restricted to trials that had used device measures (1.76, 0.82 to 3.75, P=0.15, two trials; fig 3).

Interventions involving multiple brief contacts (odds ratio 1.43, 95% confidence interval 1.18 to 1.73, P<0.001) and intensive contact support (1.24, 1.04 to 1.47, P=0.01) significantly increased the odds of participants being sufficiently physically active in line with guidelines versus controls, with moderate (I^2^=49%, P=0.02) and low (I^2^=0%, P=0.68) heterogeneity. Brief interventions (1.18, 0.73 to 1.89, P=0.51) showed no effect from the three studies included (supplementary fig 3).

Intervention group participants significantly increased their total physical activity (all intensities of physical activity combined) with a small to moderate effect found (standardised mean difference 0.32, 95% confidence interval 0.15 to 0.49, P<0.001). Substantial heterogeneity was present (I^2^=91%, P<0.001; supplementary fig 4). When stratified by self-report and device measured, a larger effect was seen for total physical activity when measured using a device (0.53, 0.14 to 0.92) compared with self-reported (0.17, 0.11 to 0.24). No significant effect was observed for time spent sedentary (mean difference −3.1 min/day, 95% confidence interval −11.8 to 5.6, P=0.48; supplementary fig 5).

Eighteen trials reported weight (all objectively measured) and there was a significant reduction of 1 kg favouring the physical activity intervention groups versus controls (mean difference −1.0 kg, 95% confidence interval −1.6 to −0.5, P<0.001) with substantial heterogeneity (I^2^=72%, P<0.001; supplementary fig 6). A sensitivity analysis was performed with Taheri and colleagues^61^ removed because this intervention included an intensive diet replacement phase and therefore had a substantially larger effect on weight than other included studies. This analysis showed that the significant effect remained (−0.7 kg, −1.1 to −0.3, P<0.001) with moderate heterogeneity (I^2^=48%, P=0.01). No intervention effect was observed for body mass index (−0.04, −0.15 to 0.07, P=0.50; supplementary fig 7).

Of the 46 studies included in the meta-analyses of MVPA (minutes per week and the proportion of participants meeting guidelines), six were considered low risk of bias, 26 had some concerns, and 14 were high risk of bias (supplementary fig 8). For measurement of the outcome, 30 studies had some concerns because they assessed MVPA using self-report measures. Most studies did not provide a prespecified analysis plan, and so they had some concerns about the selection of the reported result (n=24). High risk of bias was typically due to incomplete MVPA data at final follow-up (n=8). A sensitivity analysis comparing the studies with low risk of bias or some concerns with high risk of bias trials did not change the results for minutes per week of MVPA (mean difference 16.5 min/week, 95% confidence interval 4.2 to 28.9, P=0.009; I^2^=94%, P<0.001), or the proportion who were sufficiently physically active in line with guidance (odds ratio 1.36, 95% confidence interval 1.17 to 1.57, P<0.001; I^2^=37%, P=0.05). No evidence was found of publication bias after examining the funnel plots (supplementary figs 9-10).

## Discussion

### Principal findings

Estimates from this systematic review, which included 51 randomised controlled trials, found that physical activity interventions delivered by health professionals in primary care increased participation in MVPA in patients by an average of 14 min/week versus controls. While this size of effect might seem modest, it should be interpreted within the context that MVPA has an inverse dose-response relation with all cause mortality, therefore even small increases in physical activity are clinically important.^80^ Other systematic reviews have reported that an increase in MVPA of 2 min/day (14 min/week) is associated with an 11% reduction in all cause mortality.^81^ Intervention group participants were 33% more likely to meet guidelines for MVPA and achieved significantly more overall physical activity (total activity with all intensities combined) than controls. Multiple contacts with an intervenor, including one with a primary care health professional, are needed to increase participation in MVPA. Interventions with at least five contacts had a larger effect on self-reported minutes of MVPA than those with fewer contacts.

### Strengths and limitations of this review

This review has several strengths. It is a large, comprehensive systematic review that examined the effectiveness of physical activity interventions delivered by health professionals in primary care settings. Systematic reviews of randomised controlled trials investigating the effectiveness of such interventions on sedentary time or body weight are lacking. Our primary conclusions are based on a large sample of approximately 16 000 randomised participants worldwide, which increases generalisability. Comprehensive searches of published studies and grey literature were conducted with no restrictions on language or publication date, increasing the likelihood that all eligible trials were identified. Only five trials could not be included in the meta-analyses. Additionally, no evidence of publication bias was found. The focus of this review on MVPA allowed the findings to be put in a public health context and enables direct comparisons with the WHO physical activity guidelines to inform health policy decisions across the world.

This review also has some limitations. Stratified analyses by measurement type showed a significant increase in self-report measures but not in device based measures of MVPA. Self-report measures have been reported to have lower validity compared with device based measures and might overestimate physical activity^82^; however, this is likely to be true for the intervention and control groups, and the results do not appear to be implausibly inflated (+24 min/week, 95% confidence interval 6.3 to 42.8). While, device based measures have lower variability for validity and reliability of physical activity measurement, they also have issues with potential biases, including reactivity, incomplete data, and varying cut-off points to classify MVPA.^83^ ^84^ Additionally, fewer trials have used devices to measure MVPA, and this review included all data regardless of the method used to measure MVPA. This approach allowed all the relevant data to be processed and recommendations made based on all the available evidence.

In some trials, usual care included brief physical activity advice from a primary care health professional, which could have led to an increase in physical activity in control groups (contamination). Therefore, our findings might be an underestimation of the true effects, although we know advice alone has limited effectiveness for increasing or maintaining physical activity.^85^ Fourteen trials were at high risk of bias, but the results remained unchanged when these trials were removed from the analyses, indicating that findings are not subject to, or dependent on, trial quality. Substantial heterogeneity was found for the outcome of minutes per week of MVPA, which appears to be related to the method used to assess physical activity. Heterogeneity was substantially reduced in a subgroup analysis when data were categorised as self-report or device measured. Although this was a large review, the data from trials using a device were limited. Only 14 trials followed participants for at least two years and one trial for five years.

### Comparison with other studies

Because reviews on the effectiveness of physical activity interventions delivered by health professionals in primary care that report data on MVPA are lacking, direct comparisons with other reviews are limited. However, reviews on similar questions have reported mixed findings, with some reporting these types of interventions can be effective at increasing physical activity outcomes,^8^ ^9^ ^10^ ^13^ ^15^ while others have found limited evidence.^11^ ^14^ Our review is most closely aligned to the review by Oloo and colleagues,^15^ which reported that physical activity interventions delivered in primary care increased physical activity participation (standardised mean difference 0.11), but the Oloo review only included trials that had measured overall (total) physical activity outcomes using self-report measures, and their findings were based on data from only 14 randomised controlled trials. Of note here, Goryakin and colleagues^13^ examined the impact of primary care initiated physical activity interventions and found that increased contacts between health professionals and patients produced a larger effect than those restricted to initial referral only. However, Goryakin and colleagues included exercise referral schemes whereas the current review did not. Nevertheless, collectively these findings highlight the importance of involving health professionals in primary care settings in providing physical activity interventions to patients.

### Implications and future research

The interventions assessed in this review increased participants’ overall (total) physical activity (standardised mean difference 0.32) relative to controls at follow-up. While WHO guidelines focus on the importance of achieving 150 min/week of MVPA, they also state that all movement counts for health, regardless of intensity.^2^ Several studies have shown that light intensity physical activity can also improve health outcomes and this is particularly important in primary care for several reasons.^86^ Many patients who present to primary care health professionals might not have the means or motivation to achieve MVPA, and might be afraid to physically exert themselves to this intensity because of concerns about potential adverse outcomes (eg, older or frail patients, pregnant women, those with cardiovascular diseases or a disability). Additionally, many health professionals are reluctant to promote MVPA because they feel they lack the specialised knowledge or skills to do so, or consider it inappropriate to promote more vigorous intensity physical activity with some patient groups because of concerns about causing harm.^87^ The Global Action Plan for Physical Activity^5^ highlights the need to strengthen the training of health professionals so that competent assessments and provision of physical activity advice or counselling can be given in routine practice. Our findings can be used to reassure health professionals that interventions delivered by them in primary care can be effective in encouraging patients to be more physically active, even if this does not meet the MVPA intensity threshold recommended by WHO.

Physical activity is known to improve a wide range of health outcomes, further highlighting the importance of finding effective population based strategies to increase participation rates. This review found that patients randomised to a physical activity intervention delivered by health professionals in primary care weighed 1 kg less than control groups at follow-up. While a difference of 1 kg might appear small, this finding should be considered in the context that adults typically gain around 0.5-1 kg/year, which can contribute to the development of obesity.^88^ A small amount of weight loss is also important because the association between weight and all cause mortality is linear.^89^ Our data provide evidence that the population impacts from physical activity are likely to reduce other key health outcomes, such as weight, reducing the risk of diseases and death.

Primary care is a health context where millions of interactions between patients and health professionals take place every month. This review has highlighted the critical role that health professionals in primary care can have in supporting the public to increase their physical activity. Future research is now needed to establish the optimum number and length of contacts required to successfully initiate, and then maintain, patients’ participation in physical activity. Additionally, the effectiveness of different types of physical activity interventions and their content need to be explored in more depth. In future trials, MVPA should be measured using devices.

### Conclusions

Physical activity interventions delivered by health professionals in primary care settings appear effective in increasing participation in physical activity as measured by self-report and reducing weight in adults. Health commissioners and policy makers should consider physical activity interventions that include at least one contact with a health professional in primary care to help meet the World Health Assembly target of achieving a 15% reduction in physical inactivity by 2030.^5^

**What is already known on this topic**

Increasing population levels of physical activity is a public health priority and the World Health Assembly aims to reduce physical inactivity by 15% by 2030
Most adults visit their general practice once a year, therefore health professionals in primary care have the opportunity to routinely provide physical activity interventions to patients
Previous reviews of physical activity interventions delivered in primary care have reported mixed findings and those investigating the effectiveness of such interventions for increasing moderate to vigorous intensity physical activity (MVPA) are lacking

**What this study adds**

Physical activity interventions delivered to patients by health professionals in primary care significantly increased MVPA compared with control groups
The results are based on data from 46 randomised controlled trials involving approximately 16 000 participants worldwide
These data could help health professionals, policy makers, and healthcare commissioners make evidence based decisions about implementing physical activity interventions during consultations delivered in primary care

## Data Availability

All data relevant to the study are included in the article or uploaded as supplementary information.

## Web extra

Extra material supplied by authors

Web appendix: Supplementary materials

## Notes

Contributors: VEK and AJD designed the study, generated hypotheses and interpreted the data. VEK analysed the data with input from AJD and CDM. All authors critically reviewed the report. AC designed the searches with input from VEK and AJD. AC performed the searches and removed duplicates. VEK, AJD, CDM, HG, and JJCT screened the titles and abstracts. VEK, AJD, and AEC screened the full texts. VEK, HG, and AEC performed the data extraction. VEK and CDM completed the risk of bias assessment. VEK and AJD directly accessed and verified the underlying data reported in the manuscript. All authors had full access to all the data in the study and had final responsibility for the decision to submit for publication. VEK and AJD are the guarantors. The corresponding author attests that all listed authors meet authorship criteria and that no others meeting the criteria have been omitted.

## Notes

Dissemination to participants and related patient and public communities: We will ask our media office to issue a results press release. We plan to disseminate our findings widely through social media and with a plain language summary on the CLiMB website (https://www.lboro.ac.uk/research/climb/).

The lead author (the manuscript’s guarantor) affirms that the manuscript is an honest, accurate, and transparent account of the study being reported; that no important aspects of the study have been omitted; and that any discrepancies from the study as originally planned (and, if relevant, registered) have been explained.

Provenance and peer review: Not commissioned; externally peer reviewed.
