# Supplementary material for: Effectiveness of physical activity interventions delivered or prompted by health professionals in primary care settings: systematic review and meta-analysis of randomised controlled trials
Source: BMJ. 2022 Feb 23;376:e068465. doi: 10.1136/bmj-2021-068465 (PMC8864760; doi:10.1136/bmj-2021-068465)

Supplementary material

Search strategy (Sept 2020)
This search was devised and tested in Medline. It combines intervention (physical activity) and setting (primary care) terms with established randomised controlled trial filters.[1] No population restrictions were applied. The search was adapted for a range of datasets including bibliographic databases, both general and subject specific, alongside trial registries; conference proceedings; citation indexes and grey literature sources. The range of databases chosen to search is well balanced and tailored to the subject matter in order to provide good coverage in identification of relevant studies. Whilst several trial registries were searched, it was not possible to search WHO ICTRP at this time due to restricted access. No date limitations were included with the exception of SCI-E where the dates were restricted to the last 10 years for purposes of manageability.

[1] MEDLINE Therapy Best balance filter based on: Haynes RB, Wilczynski NL. Optimal search strategies for retrieving scientifically strong studies of diagnosis from MEDLINE: analytical survey. *BMJ* 2004; **328**:1040-2.

Additional screening information
Bibliomap, ClinicalTrial.GOV and Open Grey search results could not be uploaded to Covidence so were screened in Microsoft® Word with subsequent eligible full texts added to Covidence and duplicates removed. Google translate was used to translate full texts where required to determine eligibility.

Databases:
MEDLINE and MEDLINE In-Process: 09/09/2020
1 Exercis*.ti,ab.
2 exp exercise/ or exp physical exertion/ or exp physical fitness/
3 jog*.ti,ab.
4 (physical* adj3 (activ* or fit*)).ti,ab.
5 Aerobics.ti,ab.
6 swim*.ti,ab.
7 gym*.ti,ab.
8 sport*.ti,ab.
9 workout.ti,ab.
10 skip*.ti,ab.
11 calisthenics.ti,ab.
12 Step count*.ti,ab.
13 e-Exercise.ti,ab.
14 PACE.ti,ab.
15 pedometer.ti,ab.
16 Bicyc*.ti,ab.
17 Cycling.ti,ab.
18 (Social adj2 prescrib*).ti,ab.
19 Parkrun.ti,ab.
20 Couch to 5k.ti,ab.
21 Danc*.ti,ab.
22 or/1-21
23 (GP or general practi* or family practi*).ti,ab.
24 Family Practice/ or General Practitioners/
25 (Community adj2 health*).ti,ab.
26 (CCG or PCN or clinical commissioning group*).ti,ab.
27 ((Communit* or primary) adj3 nurs*).ti,ab.
28 exp community health nursing/ or public health nursing/
29 ((general or famil* or primary) adj3 physician).ti,ab.
30 Care home.ti,ab.
31 Community Health Services/
32 (Primary care or primary health care).ti,ab.
33 PCG.ti,ab.
34 Primary Health Care/
35 ((General or famil* or primary) adj3 doctor).ti,ab.
36 (practi* adj2 nurse).ti,ab.
37 ((general or family or primary or communit*) adj3 (physical therapist* or physiotherapist*)).ti,ab.
38 Physical Therapists/

39 or/23-38
40 39 and 22
41 (quasi adj2 (RCT or randomi?ed controlled trial)).ti,ab.
42 randomi?ed controlled trial.pt.
43 randomi?ed.ti,ab.
44 placebo.ti,ab.
45 41 or 42 or 43 or 44
46 40 and 45

PsycINFO: 09/09/2020
1 Exercis*.ti,ab.
2 Exp exercise/or exp physical activity/ or exp physical fitness
3 jog*.ti,ab.
4 (physical* adj3 (activ* or fit*)).ti,ab.
5 Aerobics.ti,ab.
6 swim*.ti,ab.
7 gym*.ti,ab.
8 sport*.ti,ab.
9 workout.ti,ab.
10 skip*.ti,ab.
11 calisthenics.ti,ab.
12 Step count*.ti,ab.
13 e-Exercise.ti,ab.
14 PACE.ti,ab.
15 pedometer.ti,ab.
16 Bicyc*.ti,ab.
17 Cycling.ti,ab.
18 (Social adj2 prescrib*).ti,ab.
19 Parkrun.ti,ab.
20 Couch to 5k.ti,ab.
21 Danc*.ti,ab.
22 or/1-21
23 (GP or general practi* or family practi*).ti,ab.
24 Family Physicians/ or General Practitioners/
25 (Community adj2 health*).ti,ab.
26 (CCG or PCN or clinical commissioning group*).ti,ab.
27 ((Communit* or primary) adj3 nurs*).ti,ab.
28 Public Health Services Nurses/
29 ((general or famil* or primary) adj3 physician).ti,ab.
30 Care home.ti,ab.
31 Community Health/ or Community Services/
32 (Primary care or primary health care).ti,ab.
33 PCG.ti,ab.
34 Primary Health Care/
35 ((General or famil* or primary) adj3 doctor).ti,ab.
36 (practi* adj2 nurse).ti,ab.
37 ((general or family or primary or communit*) adj3 (physical therapist* or physiotherapist*)).ti,ab.
38 Physical Therapists/
39 or/23-38
40 39 and 22
41 (quasi adj2 (RCT or randomi?ed controlled trial)).ti,ab.
42 random:.tw.
43 control:.tw.
44 exp treatment/
45 41 or 42 or 43 or 44
46 40 and 45

EMBASE: 10/09/2020
1 Exercis*.ti,ab.
2 exp exercise/or exp physical activity/ or exp fitness

3 jog*.ti,ab.
4 (physical* adj3 (activ* or fit*)).ti,ab.
5 Aerobics.ti,ab.
6 swim*.ti,ab.
7 gym*.ti,ab.
8 sport*.ti,ab.
9 workout.ti,ab.
10 skip*.ti,ab.
11 calisthenics.ti,ab.
12 Step count*.ti,ab.
13 e-Exercise.ti,ab.
14 PACE.ti,ab.
15 pedometer.ti,ab.
16 Bicyc*.ti,ab.
17 Cycling.ti,ab.
18 (Social adj2 prescrib*).ti,ab.
19 Parkrun.ti,ab.
20 Couch to 5k.ti,ab.
21 Danc*.ti,ab.
22 or/1-21
23 (GP or general practi* or family practi*).ti,ab.
24 General practice/
25 (Community adj2 health*).ti,ab.
26 (CCG or PCN or clinical commissioning group*).ti,ab.
27 ((Communit* or primary) adj3 nurs*).ti,ab.
28 exp community health nursing/
29 ((general or famil* or primary) adj3 physician).ti,ab.
30 Care home.ti,ab.
31 Community Health Services/
32 (Primary care or primary health care).ti,ab.
33 PCG.ti,ab.
34 Primary Health Care/
35 ((General or famil* or primary) adj3 doctor).ti,ab.
36 (practi* adj2 nurse).ti,ab.
37 ((general or family or primary or communit*) adj3 (physical therapist* or physiotherapist*)).ti,ab.
38 Physiotherapist/ (Embase)
39 or/23-38
40 39 and 22
41 (quasi adj2 (RCT or randomi?ed controlled trial)).ti,ab.
42 random:.tw.
43 placebo:.tw.
44 double-blind:.tw.
45 41 or 42 or 43 or 44
46 40 and 45

ASSIA: 11/09/2020
(ab(Exercis* OR jog* OR physical activ* OR physical fit* OR aerobics OR swim* OR gym* OR sport* OR workout OR calisthenics OR step count* OR e-exercise OR PACE OR pedometer OR bicyc* OR cycling OR social prescrib* OR parkrun* OR couch to 5k OR danc*) OR ti(Exercis* OR jog* OR physical activ* OR physical fit* OR aerobics OR swim* OR gym* OR sport* OR workout OR calisthenics OR step count* OR e-exercise OR PACE OR pedometer OR bicyc* OR cycling OR social prescrib* OR parkrun* OR couch to 5k OR danc*) OR MAINSUBJECT.EXACT("Exercise") OR MAINSUBJECT.EXACT("Physical fitness")) AND (ti(community health* OR CCG OR PCN OR clinical commissioning group* OR communit* nurs* OR primary nurs* OR general physician OR famil* physician OR primary physician OR care home OR primary care OR primary health care OR PCG OR general doctor OR famil* doctor OR primary doctor OR practi* nurse OR physical therapist* OR physiotherapist*) OR ab(community health* OR CCG OR PCN OR clinical commissioning group* OR communit* nurs* OR primary nurs* OR general physician OR famil* physician OR primary physician OR care home OR primary care OR primary health care OR PCG OR general doctor OR famil* doctor OR primary doctor OR practi* nurse OR physical therapist* OR physiotherapist*) OR MAINSUBJECT.EXACT("Family physicians") OR MAINSUBJECT.EXACT("Community nursing") OR

MAINSUBJECT.EXACT("Community health care") OR MAINSUBJECT.EXACT("Primary care") OR MAINSUBJECT.EXACT("Physical therapists")) AND (ab(randomi*ed) OR ti(randomi*ed) OR ab (placebo) OR ti (placebo) OR ti(RCT) or ab(RCT))

Database: Cinahl : 16/09/2020
S41 S34 AND S40
S40 S35 OR S36 OR S37 OR S38 OR S39
S39 PT clinical trial
S38 (MH "Treatment Outcomes")
S37 TI (randomi?ed) or AB (randomi?ed)
S36 AB (quasi n2 (RCT or randomi*ed controlled trial)
S35 TI (quasi n2 (RCT or randomi*ed controlled trial)
S34 S11 AND S33
S33 S12 OR S13 OR S14 OR S15 OR S16 OR S17 OR S18 OR S19 OR S20 OR S21 OR S22 OR S23 OR S24 OR S25 OR S26 OR S27 OR S28 OR S29 OR S30 OR S31 OR S32
S32 (MM "Physical Therapists")
S31 AB (genera or famil* or primary or communit*) n3 (physical therapist* or physiotherapist*)
S30 TI (general or famil* or primary or communit*) n3 (physical therapist* or physiotherapist*)
S29 TI (practi* n2 nurse) or AB (practi* n2 nurse)
S28 AB (General or famil* or primary) n3 doctor
S27 TI (General or famil* or primary) n3 doctor
S26 (MM "Primary Health Care")
S25 TI (PCG) or AB (PCG)
S24 TI (primary care or primary healthcare) or AB (primary care or primary healthcare)
S23 (MM "Community Health Services+")
S22 TI (Care home) or AB (Care home)
S21 AB (general or famil* or primary) n3 physician*
S20 TI (general or famil* or primary) n3 physician*
S19 (MM "Community Health Nursing+")
S18 AB (Communit* or primary) n2 nurs*)
S17 TI (Communit* or primary) n2 nurs*)
S16 TI (CCG or PCN or clinical commissioning group*) or AB (CCG or PCN or clinical commissioning group*
S15 TI (Community n2 health*) or AB (Community n2 health*)
S14 (MM "Physicians, Family")
S13 (MM "Family Practice")
S12 TI (GP or general practi* or family practi*) or AB (GP or general practi* or family practi*)
S11 S1 OR S2 OR S3 OR S4 OR S5 OR S6 OR S7 OR S8 OR S9 OR S10
S10 TX couch to 5k
S9 TI (Social n2 prescib*) or AB (Social n2 prescrib*)
S8 TI (Aerobics or swim or gym or sport* or workout or skip* or calisthenics* or Step count* or e-Exercise* or PACE or pedometer or Bicyc* or Cycling or Parkrun or Danc*) or AB (Aerobics or swim or gym or sport* or workout or skip* or calisthenics* or Step count* or e-Exercise* or PACE or pedometer or Bicyc* or Cycling or Parkrun or Danc*)
S7 AB Physical* N3 activ*
S6 TI Physical N3 activ*
S5 AB Physical* N3 fit*
S4 TI Physical* N3 fit*
S3 (MM "Physical Fitness+") OR (MM "Sports+")
S2 (MM "Exercise+")
S1 TI Exercis* or AB Exercis*

SportDiscus: 16/9/2020
S41 S34 AND S40
S40 S35 OR S36 OR S37 OR S38 OR S39
S39 PT clinical trial
S38 (MH "Treatment Outcomes")
S37 TI (randomi?ed) or AB (randomi?ed)
S36 AB (quasi n2 (RCT or randomi*ed controlled trial)
S35 TI (quasi n2 (RCT or randomi*ed controlled trial)
S34 S11 AND S33

S33 S12 OR S13 OR S14 OR S15 OR S16 OR S17 OR S18 OR S19 OR S20 OR S21 OR S22 OR S23 OR S24 OR S25 OR S26 OR S27 OR S28 OR S29 OR S30 OR S31 OR S32
S32 (MM "Physical Therapists")
S31 AB (genera or famil* or primary or communit*) n3 (physical therapist* or physiotherapist*)
S30 TI (general or famil* or primary or communit*) n3 (physical therapist* or physiotherapist*)
S29 TI (practi* n2 nurse) or AB (practi* n2 nurse)
S28 AB (General or famil* or primary) n3 doctor
S27 TI (General or famil* or primary) n3 doctor
S26 (MM "Primary Health Care")
S25 TI (PCG) or AB (PCG)
S24 TI (primary care or primary healthcare) or AB (primary care or primary healthcare)
S23 (MM "Community Health Services+")
S22 TI (Care home) or AB (Care home)
S21 AB (general or famil* or primary) n3 physician*
S20 TI (general or famil* or primary) n3 physician*
S19 (MM "Community Health Nursing+")
S18 AB (Communit* or primary) n2 nurs*)
S17 TI (Communit* or primary) n2 nurs*)
S16 TI (CCG or PCN or clinical commissioning group*) or AB (CCG or PCN or clinical commissioning group*
S15 TI (Community n2 health*) or AB (Community n2 health*)
S14 (MM "Physicians, Family")
S13 (MM "Family Practice")
S12 TI (GP or general practi* or family practi*) or AB (GP or general practi* or family practi*)
S11 S1 OR S2 OR S3 OR S4 OR S5 OR S6 OR S7 OR S8 OR S9 OR S10
S10 TX couch to 5k
S9 TI (Social n2 prescib*) or AB (Social n2 prescrib*)
S8 TI (Aerobics or swim or gym or sport* or workout or skip* or calisthenics* or Step count* or e-Exercise* or PACE or pedometer or Bicyc* or Cycling or Parkrun or Danc*) or AB (Aerobics or swim or gym or sport* or workout or skip* or calisthenics* or Step count* or e-Exercise* or PACE or pedometer or Bicyc* or Cycling or Parkrun or Danc*)
S7 AB Physical* N3 activ*
S6 TI Physical N3 activ*
S5 AB Physical* N3 fit*
S4 TI Physical* N3 fit*
S3 (MM "Physical Fitness+") OR (MM "Sports+")
S2 (MM "Exercise+")
S1 TI Exercis* or AB Exercis*

Sports Medicine and Education Index (formally Physical Education Index): 18/09/2020
(ab(Exercis* OR jog* OR physical activ* OR physical fit* OR aerobics OR swim* OR gym* OR sport* OR workout OR calisthenics OR step count* OR e-exercise OR PACE OR pedometer OR bicyc* OR cycling OR social prescrib* OR parkrun* OR couch to 5k OR danc*) OR ti(Exercis* OR jog* OR physical activ* OR physical fit* OR aerobics OR swim* OR gym* OR sport* OR workout OR calisthenics OR step count* OR e-exercise OR PACE OR pedometer OR bicyc* OR cycling OR social prescrib* OR parkrun* OR couch to 5k OR danc*) OR MAINSUBJECT.EXACT("Exercise") OR MAINSUBJECT.EXACT("Physical fitness")) AND (ti(community health* OR CCG OR PCN OR clinical commissioning group* OR communit* nurs* OR primary nurs* OR general physician OR famil* physician OR primary physician OR care home OR primary care OR primary health care OR PCG OR general doctor OR famil* doctor OR primary doctor OR practi* nurse OR physical therapist* OR physiotherapist*) OR ab(community health* OR CCG OR PCN OR clinical commissioning group* OR communit* nurs* OR primary nurs* OR general physician OR famil* physician OR primary physician OR care home OR primary care OR primary health care OR PCG OR general doctor OR famil* doctor OR primary doctor OR practi* nurse OR physical therapist* OR physiotherapist*) OR MAINSUBJECT.EXACT("Family physicians") OR MAINSUBJECT.EXACT("Community nursing") OR MAINSUBJECT.EXACT("Community health care") OR MAINSUBJECT.EXACT("Primary care") OR MAINSUBJECT.EXACT("Physical therapists")) AND (ab(randomi*ed) OR ti(randomi*ed) OR ab (placebo) or ti (placebo)) OR ti(RCT) or ab(RCT))

PEDRO (Physiotherapy Evidence Database) : 18/09/2020
(exercis* and primary care) OR (fit* and primary care OR (activ* and primary care) OR (exercis* and gp) OR (fit* and gp) OR (activ* and gp) OR (active* and communit* and health* and service*) OR (fit* and

communit* and health* and service*) OR (exercis* and communit* and health* and service*) OR (exercis* and communit* and nurs*) OR (fit* and communit* and nurs*) OR (activ* and communit* and nurs* OR (physical activ aerobic primary) OR (fit* activ aerobic primary) OR (exercis* activ aerobic primary) OR (exercise aerobic primary)

Bibliomap, EPPI CENTRE (Database of health promotion research): 18/09/2020
Freetext: fit* OR physical activit* OR exercise
Limit by study type: RCT

TRIALS REGISTRIES:
CT.gov: 23/10/2020
Other terms: “primary care” AND “exercise”
Limits: Age: Adult & Older Adult Study type: Interventional

EPPI CENTRE TRIALS REGISTRY: TROPHI: 15/09/2020
1 What type of study does this report describe?: RCT
2 Freetext (All but Authors): exercise
3 Freetext (All but Authors): "physical activit*"
4 Freetext (All but Authors): "primary care"
5 2 OR 3
6 1 AND 4 AND 5

CENTRAL: 15/09/2020
#1 ((Exercis* OR physical activ* OR physical fit*) NEAR/5 aerobic*):ti,ab,kw (Word variations have been searched)
#2 MeSH descriptor: [Exercise] explode all trees
#3 MeSH descriptor: [Physical Exertion] explode all trees
#4 MeSH descriptor: [Physical Fitness] explode all trees
#5 #1 or #2 or #3 or #4

CONFERENCE PROCEEDINGS
Conference Proceedings Citation Index – Science (CPCI-S): 15/09/2020
#8 #7 AND #3 AND #2
#7 #6 OR #1
6 #5 AND #4
5 TI= (Aerobic) OR AB =(Aerobic)
4 TI = (Exercis* OR physical activ* OR physical fit*) OR AB =(Exercis* or physical activ* or physical fit*)
3 ab=(randomi*ed) OR ti=(randomi*ed) OR ab =(placebo) OR ti =(placebo) OR ti=(RCT) or ab=(RCT)
2 ti=(community health* OR CCG OR PCN OR clinical commissioning group* OR communit* nurs* OR primary nurs* OR general physician OR famil* physician OR primary physician OR care home OR primary care OR primary health care OR PCG OR general doctor OR famil* doctor OR primary doctor OR practi* nurse OR physical therapist* OR physiotherapist*) OR ab=(community health* OR CCG OR PCN OR clinical commissioning group* OR communit* nurs* OR primary nurs* OR general physician OR famil* physician OR primary physician OR care home OR primary care OR primary health care OR PCG OR general doctor OR famil* doctor OR primary doctor OR practi* nurse OR physical therapist* OR physiotherapist*)
1 TI=(jog* OR aerobics OR swim* OR gym* OR sport* OR workout OR calisthenics OR step count* OR e-exercise OR PACE OR pedometer OR bicyc* OR cycling OR social prescrib* OR parkrun* OR couch to 5k OR danc*) OR AB=(jog* OR aerobics OR swim* OR gym* OR sport* OR workout OR calisthenics OR step count* OR e-exercise OR PACE OR pedometer OR bicyc* OR cycling OR social prescrib* OR parkrun* OR couch to 5k OR danc*)

CITATION SEARCHING:
Science Citation Index – Expanded (SCI-E): 15/09/2020
#8 #7 AND #3 AND #2 (limit years 2010-2020)
#7 #6 OR #1
6 #5 AND #4
5 TI= (Aerobic) OR AB =(Aerobic)
4 TI = (Exercis* OR physical activ* OR physical fit*) OR AB =(Exercis* or physical activ* or physical fit*)
3 ab=(randomi*ed) OR ti=(randomi*ed) OR ab =(placebo) OR ti =(placebo) OR ti=(RCT) or ab=(RCT)

2 ti=(community health* OR CCG OR PCN OR clinical commissioning group* OR communit* nurs* OR primary nurs* OR general physician OR famil* physician OR primary physician OR care home OR primary care OR primary health care OR PCG OR general doctor OR famil* doctor OR primary doctor OR practi* nurse OR physical therapist* OR physiotherapist*) OR ab=(community health* OR CCG OR PCN OR clinical commissioning group* OR communit* nurs* OR primary nurs* OR general physician OR famil* physician OR primary physician OR care home OR primary care OR primary health care OR PCG OR general doctor OR famil* doctor OR primary doctor OR practi* nurse OR physical therapist* OR physiotherapist*)
1 TI=(jog* OR aerobics OR swim* OR gym* OR sport* OR workout OR calisthenics OR step count* OR e-exercise OR PACE OR pedometer OR bicyc* OR cycling OR social prescrib* OR parkrun* OR couch to 5k OR danc*) OR AB=(jog* OR aerobics OR swim* OR gym* OR sport* OR workout OR calisthenics OR step count* OR e-exercise OR PACE OR pedometer OR bicyc* OR cycling OR social prescrib* OR parkrun* OR couch to 5k OR danc*)

GREY LITERATURE:
Open Grey: 21/09/2020
(Exercis* OR physical activ* OR physical fit*) AND (community health* OR CCG OR PCN OR clinical commissioning group* OR communit* nurs* OR primary nurs* OR general physician OR famil* physician OR primary physician OR care home OR primary care OR primary health care OR PCG OR general doctor OR famil* doctor OR primary doctor OR practi* nurse OR physical therapist* OR physiotherapist*) AND (randomi*ed OR placebo OR RCT)

PubMed updated search (01/04/2021)
GP[Title/Abstract]) OR ("General practi*"[Title/Abstract])) OR ("family practi*"[Title/Abstract])) OR (Family Practice[MeSH Terms]) OR (General practitioners[MeSH Terms]) OR (community health nursing[MeSH Terms]) OR (public health nursing[MeSH Terms]) OR (Community Health Services[MeSH Terms]) OR (Primary Health Care[MeSH Terms]) OR (Physical Therapists[MeSH Terms]) OR ("Community health*"[Title/Abstract]) OR (CCG[Title/Abstract]) OR (PCN[Title/Abstract]) OR ("clinical commissioning group*"[Title/Abstract]) OR (nurs*[Title/Abstract]) OR (physician*[Title/Abstract]) OR ("Care home"[Title/Abstract]) OR ("Primary care"[Title/Abstract]) OR ("Primary health care"[Title/Abstract]) OR ("Primary healthcare"[Title/Abstract]) OR (PCG[Title/Abstract]) OR (doctor*[Title/Abstract]) OR (physical therapist*[Title/Abstract]) OR (physiotherapist*[Title/Abstract]) Filters: Clinical Trial, Randomized Controlled Trial, from 2020/6/1 - 2021/4/1
AND
(Exercise[Title/Abstract]) OR (exercise[MeSH Terms])) OR (physical exertion[MeSH Terms])) OR (jog[Title/Abstract])) OR ("physical activ*"[Title/Abstract])) OR ("physical fit*"[Title/Abstract])) OR (Aerobics[Title/Abstract])) OR (swim*[Title/Abstract])) OR (gym[Title/Abstract])) OR (sport*[Title/Abstract])) OR (workout[Title/Abstract])) OR (skip*[Title/Abstract])) OR (calisthenics*[Title/Abstract])) OR (step count*[Title/Abstract])) OR (e-exercise[Title/Abstract])) OR (PACE[Title/Abstract])) OR (pedometer[Title/Abstract])) OR (bicyc*[Title/Abstract])) OR (cycling[Title/Abstract])) OR ("social prescrib*"[Title/Abstract])) OR (parkrun[Title/Abstract])) OR (Couch to 5k[Title/Abstract])) OR (Danc*[Title/Abstract])) OR (gymnastic*[Title/Abstract]) Filters: Clinical Trial, Randomized Controlled Trial, from 2020/6/1 - 2021/4/1

Summary table of search results prior to duplication removal

Source	Hits
ASSIA	916
CENTRAL	3193
CINAHL	3665
CPCI-S	22
CT	799
EMBASE	6247
MEDLINE AND MEDLINE IN PROGRESS	2582
PEDRO	739
PsychINFO	2647
SPORT DISCUS	312
SMEI	1638
SCI -E	1876
Open Grey	9
BIBLIOMAP, EPICENTRE	238
TROPHI, EPICENTRE	161
PubMed updated search	126
**TOTALS**	**25170**

Additional information on data synthesis methods

MVPA minutes/day were multiplied by seven and MET minutes/week[1] were multiplied by the MET value to produce minutes/week to ensure all trials were expressed in the same units. Where trials had reported moderate and vigorous intensities of physical activity separately, only the value for moderate intensity physical activity was included. If the percentage of the number of participants meeting the guidance amount of MVPA was reported, these data were divided by 100 and multiplied by the number of participants in the trial group to derive the number of participants sufficiently physically active according to guidance used in the individual RCTs. Other MVPA measures including kilocalories, number of MVPA episodes, and ordinal scores of MVPA were excluded. Standard deviations (SDs) were calculated for studies reporting only CIs or standard errors (SEs) in RevMan. Where mean change in MVPA between baseline and follow-up was not reported, it was calculated by subtracting the follow-up mean from the baseline mean. The standard deviation of mean change was calculated using a standardised formula inputting a correlation coefficient of 0.31 for self-reported MVPA data, 0.6 for device-measures of MVPA and sedentary time,[2] 0.96 for weight and BMI.[3] Medians[4,5] with calculated SDs[6] and geometric means[7] were included where authors did not respond to requests for arithmetic means and sensitivity analyses were conducted to examine the effect (not pre-specified).

Where studies reported multiple follow-up points, data from the last follow-up were included in analyses. If studies reported data from both self-report and device-measures, only device-measured data were included in analyses. If there was more than one intervention group, the number of comparison participants was divided by the number of intervention groups and each intervention was analysed separately. In cluster randomised trials, clustering was accounted for by dividing the number of participants in each trial group by the design effect [1 + (M-1) x ICC]. Where the ICC was not reported, a value from a similar included study was used.[8–13] Heterogeneity was quantified using the I^2 statistic with roughly 75-100% considered as high, 50-90% substantial, 30-60% moderate and 0-40% might not be important according to the Cochrane Handbook.[14]

References

1 Craig CL, Marshall AL, Sjostrom M, *et al.* International physical activity questionnaire: 12-country reliability and validity. *Med Sci Sports Exerc* 2003; 35: 1381–95.

2 Alonso-Domínguez R, Patino-Alonso MC, Sánchez-Aguadero N, García-Ortiz L, Recio-Rodríguez JI, Gómez-Marcos MA. Effect of a multifactorial intervention on the increase in physical activity in subjects with type 2 diabetes mellitus: a randomized clinical trial (EMID study). *Eur J Cardiovasc Nurs* 2019; 18: 399–409.

3 Jolly K, Lewis A, Beach J, *et al.* Comparison of range of commercial or primary care led weight reduction programmes with minimal intervention control for weight loss in obesity: Lighten Up randomised controlled trial. *Bmj* 2011; 343: d6500.

4 Lawton BA, Rose SB, Elley CR, Dowell AC, Fenton A, Moyes SA. Exercise on prescription for women aged 40-74 recruited through primary care: two year randomised controlled trial. *Bmj* 2008; 337: a2509.

5 Mitchell KE, Warrington V, Sewell L, *et al.* A randomised controlled trial of a self-management programme of activity coping and education-space for COPD: Impact on physical activity at 6 weeks. *Am J Respir Crit Care Med* 2013; 187: A5952.

6 Wan X, Wang W, Liu J, Tong T. Estimating the sample mean and standard deviation from the sample size, median, range and/or interquartile range. *BMC Med Res Methodol* 2014; 14: 1–13.

7 Hardeman W, Mitchell J, Pears S, *et al.* Evaluation of a very brief pedometer-based physical activity intervention delivered in NHS Health Checks in England: the VBI randomised controlled trial. *PLoS Med* 2020; 17: e1003046.

8 Grandes G, Sanchez A, Montoya I, Ortega Sanchez-Pinilla R, Torcal J, Group P. Two-year longitudinal analysis of a cluster randomized trial of physical activity promotion by general practitioners. *PLoS One*; 6: e18363.

9 Hesselink AE, Bilo HJG, Rutten GEH, *et al.* Effectiveness of an intervention in primary care to prevent type 2 diabetes in people with impaired fasting glucose, a cluster-randomised controlled trial. *Diabetologia* 2013; 56: S165.

10 Kloek CJJ, Bossen D, Spreeuwenberg PM, Dekker J, de Bakker DH, Veenhof C. Effectiveness of a blended physical therapist intervention in people with hip osteoarthritis, knee osteoarthritis, or both: a cluster-randomized controlled trial. *Phys Ther* 2018; 98: 560–70.

11 Elley C, Kerse N, Arroll B, Robinson E. Effectiveness of counselling patients on physical activity in general practice: Cluster randomised controlled trial. *BMJ Br Med J* 2003; 326: 793–8.

12 Westland H, Schuurmans MJ, Bos-Touwen ID, *et al*. Effectiveness of the nurse-led Activate intervention in patients at risk of cardiovascular disease in primary care: a cluster-randomised controlled trial. *Eur J Cardiovasc Nurs* 2020; 19: 721–31.

13 Yates T, Edwardson C, Henson J, *et al.* Walking Away from Type 2 diabetes: a cluster randomized controlled trial. *Diabet Med* 2017; 34: 698-707.

14 Higgins JPT, Thomas J, Chandler J, Cumpston M, Li T, Page MJ WV (editors). Cochrane Handbook for Systematic Reviews of Interventions version 6.2 (updated February 2021). 2021.

Supplementary Table 1: Intervention details of included trials

Study (year)	Intervention summary	Number of intervention contacts	Adherence to intervention	Comparison group summary
**Aittasalo (2006) Finland**	PREX: Prescription-based counselling by physician including written prex (optional self-monitoring log, referral to other healthcare professionals). MON: self-monitoring (pedometer & log) & feedback from physiotherapist on 5-day recordings via mail.	1	Not reported.	Usual care.
**Alonso-Dominguez (2019) Spain**	Effectiveness of a multifactorial intervention in diabetes (EMID): nurse PA & diet counselling with leaflet (10 mins: 5 mins PA + 5 mins diet), 5 nurse-guided group 4 km low/moderate intensity walks with 10 min warm-up and 10 min stretching after (once per week for 5 weeks), smartphone & EVIDENT II app (counts steps, PA log, advice) for 3 months, and diet workshop of 1 hour.	7	Not reported.	Usual care: standardised 10 min nurse PA and diet counselling with leaflet.
**Apinaniz (2019) Spain**	PA and diet health advice plus AKTIDIET smartphone app which included an aerobic exercise program & muscle training, record of food intake, videos of exercise, advice via text message once per day for first month then twice per week until 6 months.	1	Not reported.	Usual care: health advice on PA and diet which was also provided in writing.
**Arija (2018) Spain**	9-month nurse & PA specialist supervised walking group of 396 METs/min/week over 120 min, in 2 sessions of 60 min (~5 km) with socio-cultural activities monthly.	72	Not reported.	Standard clinical care by the healthcare professionals
**Carroll (2010) US**	4 computerized personalised feedback reports based on PA surveys completed via mail (0, 1, 3 & 6 months) and included a PA prescription which they took to next physician appointment to discuss.	1	73% received the intervention materials and 87% reported reading all or most of the materials.	Identical procedures except questions were regarding preventive tests. The feedback reports contained information on recommended preventive tests and questions to ask their provider.
**Cheng (2018) US**	SUSTAIN (Systemic Use of STroke Averting INterventions): Delivered by nurse practitioners or physician assistant (care managers). Two 1 hour NP Clinic visits (1 month & 10 months) where content from group clinics is reinforced. Given report card on risk factors to take to future primary care provider visits. Blood pressure monitors & telephone care-coordinated calls started 1 week after discharge and between group and individual clinics with care manager (x5) & three 2 hour group clinics (2, 5 & 7 months) focused on stroke education, self-management including PA. After each group clinic, brief one-to-one sessions with care manager reinforced content individually and solved problems.	10	56% attended at least 2 group clinics, 28% attended none.	Usual care: mailed the American Heart Association brochure "Controlling Your Risk Factors: Our Guide to Reducing Your Risk of Heart Attack and Stroke." At least 1 scheduled appointment to the outpatient neurology clinic, followed by a plan to rapidly

				transition care to a primary care provider.
**Clapperton (2020) Trinidad**	Structured physician delivered counselling on PA, exercise prescription and follow-up phone call by physician 2 months later.	2	Not reported.	Usual care from any doctor not participating the study.
**Driehuis (2012) The Netherlands**	Groningen Overweight And Lifestyle study (GOAL): Tailored lifestyle counselling by nurse practitioner on PA and diet guided by a standardised computerised software programme for 3 years. 1st year: 4 individual counselling meetings (1, 2, 3 and 8 months) and 1 feedback consultation by phone (5 months) including pedometer & feedback. 2nd & 3rd years: 1 meeting and 2 feedback calls per year with nurse.	11	Not reported.	Usual care: 1 GP 10 min consultation (discussed baseline measures).
**Dubbert (2008) US**	Counselling linked with primary care visits on home-based walking and 'strength exercise' (based on National Institute on Aging materials) with nurse at baseline, 1 month & 5 months. Recorded exercise on monthly calendars and received brief follow-up calls from nurse (3-5 mins, 1 week after visits) and automated motivational messages (once every 3-4 weeks). Fall prevention safety brochures and bimonthly newsletters throughout 10-month trial.	6	Not reported.	Discussion of their choice of health education topics with nurse at baseline, 1 & 5 months (if exercise was chosen then only the brochure's summary was advised- no goal setting, instructions or equipment). Fall prevention safety brochures and bimonthly newsletters throughout 10-month trial.
**Duijzer (2017) The Netherlands**	SLIMMER: 38 sports lessons with primary care physiotherapist (plus dietary intervention of 6 individual consultations with a dietician & 1 group session with the aim to adopt a healthy dietary pattern). Maintenance phase at 10 months lasting up to 3 months: sports clinics at local sports club, concluding meetings with dietician and physiotherapist, and a return session with dietician, physiotherapist and the PA group.	45	Not reported.	Usual care and written materials on diet and PA.
**Dutton (2006) US**	Physician delivered tailored weight loss intervention to promote PA. 6 monthly 15 min physician counselling visits (physicians were given protocols tailored to the visits), patients received summary handouts from physicians at each visit.	6	Not reported.	Usual care for obesity management.
**Elley (2003) New Zealand**	Waikato Heart, Health & Activity Study/Green prescription programme: GP gave oral & written advice (green prescription) on PA during usual consultation and exercise specialists continued to support via telephone (at least 3 calls lasting 10-20 mins over 3 months using motivational interviewing) & post (quarterly newsletters). GP staff encouraged to feedback to participants at subsequent visits.	4	Not reported.	Usual care delivered by GPs. Offered intervention at the end of the study.
**Fortier (2011) Canada**	The Physical Activity Counselling Trial (PAC): 2-4 mins brief PA counselling with physician and 6 sessions with PA counsellor in primary care over 3 months: 3 in person (60+40+40 mins) and 3 calls (20 mins each). Written goal sheet/action plan	7	81.7% attended all evaluation	Brief PA counselling (2-4 mins) with physician.

	after in-person sessions with self-monitoring sheet. Progress report filed in patient's chart halfway through. Healthcare practitioner encouragement letter at week 10.		visits (both groups).	
**Garcia-Ortiz (2018) Spain**	EVIDENT II: Counselling on PA & Mediterranean diet with research nurse (30 mins), leaflet and 15 min training session on smartphone app which included a log for diet & exercise (use for 3 months), daily report and plan for following days. Follow-up 1 week after to check app was being used correctly.	3	56.8% had high app adherence, 28.2% used it for <1 month.	Brief counselling (30 mins) on PA and Mediterranean diet by research nurse and leaflets.
**Goldstein (1999) US**	The Physically Active for Life (PAL) project: brief physician-delivered PA counselling based on the Transtheoretical Model of Change and social learning theory. Researcher interviewed patient before seeing physician to establish motivational readiness for PA (6 mins). Physician Stage-matched counselling with written exercise prescription (5 mins) and manual. Follow-up with physician to discuss activity counselling (within 4 weeks of initial appointment) and new exercise prescription. Newsletter mailings (5x monthly mailings after follow-up).	2	Not reported.	Usual care delivered by physician.
**Gomez-Huelgas (2015) Spain**	Multidisciplinary intervention in primary care (IMAP): 3-year trial of a dietary intervention and PA programme (home-based). 27 visits (9 medical and 18 nursing) with 6 visits in first 3 months then medical every 3 months during first year then every 6 months. Health assessment was 15 min. First 6 nursing visits were 30 mins then the rest were conducted in groups lasting 30 mins. A visit for individual nursing was every 6 months receiving a total of 11 individual and 7 other group visits.	27	Participants reported similar adherence to diet, PA and quality of life at baseline.	Usual care: 4 medical consultations and nurse visits (10 mins) on an annual basis.
**Grandes (2011) Spain**	Experimental Programme for Physical activity promotion (PEPAF): Physician advice structured using web-based software and educational materials. 15 min appointment to prescribe a PA plan.	1	Not reported.	Usual care: physicians assessed patients' PA but delayed any intervention until the end of the study.
**Hall (2011) & Morey (2009) US**	Project LIFE: Multicomponent PA counselling. One-time clinical endorsement by physician, initial brief assessment in person (counselling, resistance bands, exercise poster & workbook, pedometer), every other week for 6 weeks and then monthly telephone counselling by lifestyle counsellor averaging 18 mins (1-year duration). Monthly automated telephone messages from primary care provider, tailored report mailed quarterly. Quarterly tailored mailings of PA progress.	15	Not reported.	Usual care from primary care providers.
**Harari (2008) UK**	Patients completed Health Risk Appraisal for Older Persons (HRA-O) questionnaire including PA assessment via mail. The results were uploaded to primary care provider database, a report was sent to the patient with materials, a 1-page report sent to the provider and attached to patient record. How the feedback was addressed was left up to the patient and provider.	1	Not reported.	Usual care and sent health risk assessment questionnaire after the study.

**Hardeman (2020) UK**	Step it up: Very brief pedometer intervention delivered by practitioner after NHS health check. 5 min face-to-face discussion, provision of pedometer, step chart for self-monitoring & booklet.	1	Not reported.	NHS health check only.
**Harris (2012) Australia**	Health Improvement & prevention study (HIPS): Health check with GP & practice nurse who provided brief lifestyle counselling then referred to lifestyle modification program if at high risk of CVD- visit with dietician/exercise specialist and group education programme CHANGE for HIPS ran by intervention officer: 4x 1.5 hours over 3 months then 2 follow-up sessions at 6- & 9-months including education, PA (20-30 mins walking/resistance exercise) and encouraged to use pedometer between sessions. Follow-up visit with GP 10-12 weeks later and follow-up 8-9 weeks after.	9	30.5% of intervention patients attended the group program.	Usual care for their risk factors, including routine pharmacological management.
**Harris (2018 PACE-Lift) UK**	4 individually tailored PA consultations with primary care nurse over 3 months. These included a pedometer, PA feedback, counselling, PACE-Lift patient handbook, step-count diary and walking/PA plan.	4	86% attended all nurse sessions.	Usual care.
**Harris (2018 PACE-UP) UK**	Nurse group: 12-week pedometer-based walking intervention delivered in 3 practice nurse PA consultations. Components: pedometer, individualised step-count/PA goals, patient handbook,	3	74% attended all nurse sessions, 81% sent PA diaries back with step-counts.	Usual PA and offered feedback, a pedometer, handbook and diary at the end of the trial.
**Hellgren (2020) Sweden**	Basic intervention: Information & brochures, offered free blood glucose test, telephone number for personal nurse support, PA prescription to see physiotherapist for advice, step-counter. Intensive: brief intervention plus 8 group PA sessions (6 in 6 months then 2 during following 6 months). Nurse, physiotherapist & nutritionist chaired the sessions. 2 additional groups sessions in year 2. 3rd year: telephone call every 3rd month. Groups were merged into one due to limited numbers and similar results.	16	Not reported.	Usual care: information about the participant's condition was given & brochures on diet & PA.
**Hesselink (2013) The Netherlands**	Road map towards diabetes prevention (RM): diagnosis of impaired fasting glucose by GP, motivational interviewing by practice nurse then 3 consultations in first 3 months followed by a consultation every 3 months. Referred to dietician, physiotherapist and/or local sports activities at nurse’s discretion. 12-month duration.	7	52% of both groups attended 5 or more consultations.	Usual care.
**Huebschmann (2018) US**	Be Active programme: PA tracker (Fitbit), six 25 min counselling calls with clinic staff coach (Bachelors/Masters), and three 20 min in person clinician visits for safety monitoring.	9	Not reported.	Enhanced usual care: printed PA materials & 3 monthly mailings on diet and other diabetes topics.

**Jimmy (2005) Switzerland**	Advice plus: feedback from practitioner on current stage of change related to PA level (2-10 mins), a stage specific leaflet and offered patient a counselling session (45 mins) with a PA specialist at a quarter of the usual price with 3- & 12-week phone reminders.	5	42% chose to take part in the counselling session.	Feedback from practitioner on current stage of change related to PA level and recommendation.
**Jolly (2017) UK**	Telephone health coaching intervention delivered by nurses to support self-management in relation to smoking, PA, medication management & action planning with supporting written documents, a pedometer, and self-monitoring diary. 4 sessions (1x 35-60 mins then 3x 15-20 mins) over 11 weeks, postal information at weeks 16 & 24. Baseline assessments by research nurse.	4	75.4% received all four calls.	Usual care: standard COPD information leaflet about self-management.
**Kloek (2018) The Netherlands**	e-Exercise: 3-month intervention where 5 face-to-face physical therapy sessions with primary care physiotherapist were integrated with an online application consisting of graded activity, exercise, and information modules. Weekly emails reminders and automatic tailored feedback.	5	81.1% were classified as adherent.	Usual physiotherapy.
**Lawton (2008) New Zealand**	Green prescription: Brief PA motivational interviewing by nurse 7-13 mins with 6-month follow-up interview (counselling) with primary care nurse (30 mins) & monthly telephone counselling over 9 months by exercise facilitator (average of 5 calls of 15 mins each).	7	Not reported.	Usual care.
**Migneault (2012) US**	Telephone-Linked-Care: Culturally adapted to African American adults, automated computer-based telephone counselling to improve adherence to medication, diet and PA guidelines. Before randomisation: Health education study visit (in-home) by research assistants with manual, pedometer & scales plus a 20 min education session based on manual. After randomisation: blood pressure monitor and training on telephone system. 1 call per week for 32 weeks. Included at home measurements (pedometer, sphygmomanometer, scales). Summary data printouts were provided regularly to primary care practitioner.	2	15% did not call the system, mean number of completed calls was 9 out of 32. 45.6% completed at least 1 PA module call and 49% of PA calls were completed overall.	Education only - Health education study visit (in-home) by research assistants with manual, pedometer & scales plus 20 min education session based on manual.
**Mitchell (2013) UK**	SPACE FOR COPD: 6-week self-management programme of activity, coping & education. Structured around a 176-page workbook containing educational materials and a home exercise programme (daily walking programme & resistance training). Introduced to programme by primary care physiotherapist (30-45 mins) including motivational interviewing 2 telephone calls at weeks 2 & 4 (10 mins each).	3	Not reported.	Usual care for COPD management.

**Moreno (2019) Spain**	Spanish Diabetes self-management programme (SDSMP): 6 weekly structured peer-to-peer workshops of 2.5 hours (once a week for 6 weeks) delivered by two leaders (one with diabetes/caregiver and one healthcare professional). The sessions were aimed at enabling participants to acquire knowledge and skills with a strong emphasis on healthier lifestyles including PA.	6	80% attended at least 4 sessions.	Usual care by primary healthcare professional and education on diabetes is usually offered by the nurse on an individual basis.
**Morey (2012) US**	Enhance fitness trial: 12-month, home-based multicomponent PA counselling program including one in-person baseline counselling session with trained counsellor, PA prescription and pedometer. Regular telephone counselling every 2 weeks for 6 weeks then monthly. Physician endorsement in clinic at next clinic visit with monthly automated encouragement from primary care provider via telephone, and customised mailed materials.	16	Not reported.	Usual care: consultation in medical centre and weight management program (MOVE). Participant decided whether to engage or not.
**Pears (2016) UK**	3 Very Brief Interventions delivered after NHS preventative health check in primary care by primary care nurse or health care assistants: Motivational- composed of 12 behaviour change techniques (BCTs) and included PA counselling, diary & booklet to set goals, make action plans and self-monitor PA. Pedometer- 7 BCTs and included a pedometer, recommendations based on 10,000 steps per day, step chart and booklet. Combined- 15 BCTs containing all the components of Motivational and Pedometer interventions.	1	Not reported.	Usual care: NHS preventative health check in primary care.
**Pinto (2002) US**	Fully automated PA counselling system (telephone-linked communication, TLC-PA) that was delivered via telephone. 1 home visit to train patients on the system by researcher. Participants were asked to call TLC every week for 1st 3 months then biweekly (10 mins per call). Reported pedometer readings during calls. Printed reports were sent monthly to participant & physician to facilitate integration of TLC-PA with the user's medical care, with the expectation that the reports would prompt the physician to reinforce the value of PA during a medical visit.	1	The mean number of calls to TLC-PA was 5.6 (SD=7.0). 33% did not call the system at all, 33% made 1-5 calls, 13% 6-10, 17% 11-20, 4% >20.	Comparison group (TLC-Eat) received an automated intervention promoting healthy eating, which was also delivered via telephone.
**Pinto (2005) US**	PAL2 (Physical Activity for Life 2): brief advice to exercise from a clinician (3-5 mins) supplemented by 3 face-to-face PA counselling sessions with health educator 0, 1 & 3 months (30-45 mins), PA prescriptions, 12 telephone-based counselling calls (weekly for 1st 3 months lasting 10-15 mins) and 12 PA tip sheets sent by mail at the same time as the counselling calls by health educators (research staff).	16	100% attended the 1st counselling session, 83% the 2nd, 78% the 3rd. 86% of the calls were delivered.	Brief advice (3-5 mins) from a clinician.

**Reed (2008)<br>US**	Physicians and nurses delivered either 1) Counselling (6A's & PA prescription) & educational map illustrating all accessible recreational facilities within a 2-mile radius or 2) Counselling only.	1	Not reported.	Usual care.
**Richardson (2007)<br>US**	PA education information from physician of 3-5 mins either 1) verbal 2) written (brochure) 3) combination counselling both verbal and brochure. All participants received a pedometer and record calendar for 4 weeks.	1	Not reported.	Usual care: pedometer and chart for 4 weeks only (no PA counselling from physician).
**Schillinger (2009)<br>US**	The Improving Diabetes Efforts Across Language and Literacy (IDEALL) project: Group 1) Interactive, weekly automated (pre-recorded) telephone calls over 39 weeks which took 6-10 mins for patient to complete. Self-management support with nurse follow-up call if needed (ATSM). Group 2) Monthly group medical visits with primary care physician and health educator facilitation lasting 90 mins each over 9 months (GMV) where PA was a component of self-care.	9	94% completed ≥1 ATSM call, mean number of calls completed was 21.9 of 39 delivered. 69% attended ≥1 GMV, mean number attended was 4.8 out of 9 offered.	Usual care.
**Steptoe (1999)<br>UK**	Change of Heart Study: Brief behavioural counselling, based on the stage of change model, carried out by practice nurses to reduce smoking and dietary fat intake and to increase regular physical activity. 3 sessions (patients with 2 risk factors) or 2 sessions (patients with 1 risk factor) of 20 mins counselling with nurse. Nurse called 1-2 times between sessions to consolidate and encourage.	3, 4 or 5	90.2% attended ≥1 counselling session, 72.8% attended 2, 55.7% attended 3.	Usual care: information provision & advice.
**Taheri (2020)<br>Qatar**	Diabetes Intervention Accentuating Diet and Enhancing Metabolism (DIADEM-I): intensive lifestyle intervention including a total diet replacement phase (12 weeks), gradual food reintroduction combined with PA support phase (12 weeks) and a maintenance phase involving structured lifestyle support (6 months). Also had a wrist-worn accelerometer and directed to apps for monitoring. Saw physician at baseline and then every 3 months. Saw dietitians & personal trainers every 2 weeks for 6 months then attended the clinic once/month (1 hour per visit).	22	67% of all possible visits were attended.	Usual medical diabetes care according to clinical guidelines.
**Tiessen (2013)<br>The Netherlands**	SPRING study (Self-monitoring and Prevention of Risk factors by Nurse practitioners in the region of Groningen): Standard treatment plus pro-active intensive counselling with feedback on behaviours and self-monitoring (pedometer with step diary, weighing scale and/ or blood pressure device). At least 3 nurse	6	The mean number of visits was 4.9 (SD 2.2) with	Usual care by practice nurse: One single advice session & standard information leaflet from Dutch GP society.

	follow-ups at monthly intervals and then at 3-month intervals. 1 year study. Initial visit was 20 mins and follow-ups were adapted to patient preference.		a median duration of 27 mins/visit.	
**Valve (2013) Finland**	LINDA (Finnish expression "brief intervention for ladies"): (counselling on sexual health and contraception from the study nurses as part of the vaccination trial) plus 20-minute individualized lifestyle counselling session followed by further support at the six-monthly follow-up visits of the vaccination trial, in total for 1.5–2.5 years. Follow-up included brief counselling in a supportive manner and were 3-5 sessions depending on how long the patient had left in vaccination trial. The LINDA solution-focused brief therapy intervention focused on healthy physical activity, and dietary and sleeping behaviours, based on the needs and interests of the participants.	4, 5 or 6	47% chose to discuss PA.	Counselling on sexual health and contraception from the study nurses as part of the vaccination trial & standard lifestyle counselling.
**Van der Weegan (2015) The Netherlands**	Group 1) Tool+SSP: monitoring & feedback using an accelerometer linked to a smartphone and webserver (It's Life! Tool) and self-management support programme (SSP) consisting of visits with practice nurse 3-4 times for PA counselling in a 4–6-month period (20 mins) and materials. Group 2) SSP only.	4	Tool+SSP: 80% received a minimum of 3 consultations spread over at least 3 months. SSP only: 86%.	Usual care.
**Van Sluijs (2005) The Netherlands**	Physician-based assessment and counselling for exercise (PACE): 10 min consultation offering advice based on PACE materials with GP or nurse, follow-up visit 4 weeks after with provider and PA counsellor calls at 2 weeks and 12 weeks.	4	Not reported.	Usual care: PA advice in 10 min consultation with provider.
**Vermunt (2012) The Netherlands**	Active Prevention in High Risk individuals Of Diabetes Type 2 in and around Eindhoven (APHRODITE) study: lifestyle counselling from the nurse practitioner and the general practitioner in 11 consultations of 20 mins over 2.5 years, 5 group meetings of 1 hour each to give more detailed information on diet and exercise conducted by trained dietitians (meetings 1, 2, 4, and 5) and physiotherapists (meeting 3), and a 1 hour personal consultation with the dietitian where dietary intake according to a 3-day food record was discussed, and suggestions were given for improvement.	17	Attendance at individual consultations ranged from 80-89% with GP and 86-97% with nurse.	Usual care consisted of oral and written information about type 2 diabetes from GP.
**Volger (2013) US**	Practice-based Opportunities for Weight Reduction at the University of Pennsylvania (POWER-UP): Group 1) Brief Lifestyle Counselling: quarterly primary care provider visits (5-7 mins) plus monthly lifestyle counselling sessions about behavioural weight control with medical assistant (10-15 mins). Group 2)	32	2-year attendance rates for brief: 71.8±28.6% &	Usual Care: quarterly primary care provider visits that included education on diet and exercise (5-7 mins).

	Enhanced Brief lifestyle counselling: same components as group 1 plus a choice of meal replacements or weight loss medication.		enhanced: 76.7±27%.	
**Westland (2020) The Netherlands**	Activate Intervention: four primary care nurse-led physical activity behaviour change consultations within a 3-month period (weeks 1, 2, 6 & 12) with a duration of 30 mins then three sessions of 20 mins plus a workbook.	4	74% patients attended ≥3 consultations.	Usual care according to the Dutch guidelines for CVD risk management.
**Writing Group for the Activity Counselling Trial Research Group (2001) US**	Group 1) Staff Assistance: physician advice (2-4 mins) & written educational materials, health educator showed 17 min video on ACT in a 30-40 min session, interactive mail (monthly newsletter with mail back card for reporting weekly PA, educator replied), pedometer with log, health educator behavioural counselling (10-15 mins) at naturally occurring physician visits, and phone call after 1 week (5-10 mins). Group 2) Staff Counselling: all assistance group plus regular (~15 calls) phone counselling (one per week for 2 weeks, biweekly for 6 weeks then monthly for 1st year then negotiated frequency in 2nd year) & weekly behavioural classes (1 hour each).	3 & 44	Assistance: mean 22 contacts totalling 3 hours. Counselling: mean 44 contacts totalling 9 hours for women & 38 totally 6 hours for men.	Usual care: physician advice & written educational materials consistent with national recommendations for physician counselling.
**Yates (2017) UK**	Walking away from type 2 diabetes: 3-year study with one 3 hour structured educational programme designed to promote physical activity and a healthy lifestyle delivered by trained health educators (primary care health professionals), provision of a pedometer, annual group-based maintenance workshops with health educators (two workshops of 2-3 hours each) and received telephone contact halfway between annual sessions to maintain motivation (x3 calls).	6	77% attended initial session, 59% attended initial and ≥1 refresher, 41% attended all sessions.	Sent standardized booklet detailing information on Type 2 diabetes mellitus risk and how PA and lifestyle change can be used to prevent or delay the disease.

Supplementary Figure 1: MVPA minutes/week by country

Study or Subgroup	Intervention Mean	Intervention SD	Intervention Total	Control Mean	Control SD	Control Total	Weight	Mean Difference IV, Random, 95% CI
**10.10.1 USA**								
Huebschmann 2018*	20.6	49.5	26	22.6	60	22	4.1%	-2.00 [-33.47, 29.47]
Dubbert 2008**	46.8	36.2	90	42.5	33.8	88	6.3%	4.30 [-5.99, 14.59]
Hall 2011	9	86.4	199	4.2	85.3	199	5.7%	4.80 [-12.07, 21.67]
Carroll 2010¶	114.6	350.2	113	71.5	451.6	128	0.9%	43.10 [-58.34, 144.54]
Pinto 2005*‖	62.8	12.1	49	16.6	12.8	44	6.6%	46.20 [41.12, 51.28]
Morey 2012*	60.2	151.1	180	-2.7	191.5	122	3.3%	62.90 [22.38, 103.42]
Schillinger 2009 (Group)*	35.5	401.7	99	-33.6	401.7	53	0.5%	69.10 [-64.90, 203.10]
Schillinger 2009 (Telephone)*	119	399.4	101	-4.9	399.4	53	0.5%	123.90 [-8.88, 256.68]
**Subtotal (95% CI)**			**857**			**709**	**27.8%**	**27.21 [2.66, 51.76]**
Heterogeneity: Tau² = 743.01; Chi² = 74.30, df = 7 (P < 0.00001); I² = 91%								
Test for overall effect: Z = 2.17 (P = 0.03)								
**10.10.2 Netherlands**								
van Sluijs 2005¶	68.8	597.2	110	108.7	561.5	127	0.4%	-39.90 [-188.20, 108.40]
Driehuis 2012	7.9	443.7	225	43.1	495.4	232	1.2%	-35.20 [-121.37, 50.97]
Kloek 2018¶	-6.1	97.3	108	9.9	99.3	98	4.6%	-16.00 [-42.89, 10.89]
Duijzer 2017	17.9	684.4	117	28.2	609.1	122	0.4%	-10.30 [-174.80, 154.20]
Vermunt 2012	-20.2	420.9	543	-16.1	345.3	522	2.8%	-4.10 [-50.26, 42.06]
van der Weegen 2015 (SSP)¶*	-14.9	183.4	54	-12.1	123	28	1.7%	-2.80 [-69.65, 64.05]
Westland 2020¶*‖	46.9	143.9	62	7	154	57	2.3%	39.90 [-13.78, 93.58]
van der Weegan 2015 (Tool+SSP)¶*	66.7	135.9	53	-12.1	123	28	2.1%	78.80 [20.37, 137.23]
**Subtotal (95% CI)**			**1272**			**1214**	**15.5%**	**7.29 [-20.34, 34.92]**
Heterogeneity: Tau² = 542.90; Chi² = 11.40, df = 7 (P = 0.12); I² = 39%								
Test for overall effect: Z = 0.52 (P = 0.60)								
**10.10.3 UK**								
Hardeman 2020**†	77.3	35.3	417	76.7	34.2	442	6.6%	0.60 [-4.05, 5.25]
Yates 2017¶	-17.7	88.5	321	-27	105.9	292	5.8%	9.30 [-6.23, 24.83]
Harris 2018 (PACE-UP)¶	25.3	135.2	346	7.6	110.2	338	5.5%	17.70 [-0.77, 36.17]
Harris 2018 (PACE-Lift)¶	6.5	128.2	150	-15	134.5	148	4.3%	21.50 [-8.34, 51.34]
Jolly 2017	-15.9	205.9	289	-51.2	218.7	288	3.8%	35.30 [0.64, 69.96]
**Subtotal (95% CI)**			**1523**			**1508**	**26.0%**	**11.04 [-0.18, 22.25]**
Heterogeneity: Tau² = 79.27; Chi² = 8.90, df = 4 (P = 0.06); I² = 55%								
Test for overall effect: Z = 1.93 (P = 0.05)								
**10.10.4 Spain**								
Garcia-Ortiz 2018*	-93	259.1	415	-51	239.2	418	3.9%	-42.00 [-75.87, -8.13]
Grandes 2011¶*‖	148.8	743.7	633	140	721.2	593	1.3%	8.80 [-73.21, 90.81]
Moreno 2019	319	466.6	221	307	439.7	228	1.2%	12.00 [-71.92, 95.92]
Alonso-Dominguez 2019	63.3	355.2	94	-30.8	450.2	91	0.7%	94.10 [-23.00, 211.20]
Arija 2018	112.3	426.3	152	-51.3	307.5	55	0.8%	163.60 [57.78, 269.42]
**Subtotal (95% CI)**			**1515**			**1385**	**7.8%**	**36.67 [-35.35, 108.69]**
Heterogeneity: Tau² = 4870.68; Chi² = 17.27, df = 4 (P = 0.002); I² = 77%								
Test for overall effect: Z = 1.00 (P = 0.32)								
**10.10.5 Other**								
Taheri 2020	-10.8	210.5	79	48.1	245.7	79	1.6%	-58.90 [-130.25, 12.45]
Fortier 2011*	-1.3	4.2	61	-2	4.1	59	6.7%	0.70 [-0.79, 2.19]
Aittasalo 2006 (MON)¶	4	54.5	58	3.2	59.1	33	4.8%	0.80 [-23.76, 25.36]
Aittasalo 2006 (PREX)¶	22.2	71.1	117	3.2	59.1	33	4.9%	19.00 [-4.93, 42.93]
Elley 2003¶*	54.6	142.6	226	16.8	113.5	214	4.9%	37.80 [13.78, 61.82]
**Subtotal (95% CI)**			**541**			**418**	**22.9%**	**9.06 [-8.68, 26.80]**
Heterogeneity: Tau² = 246.71; Chi² = 14.03, df = 4 (P = 0.007); I² = 71%								
Test for overall effect: Z = 1.00 (P = 0.32)								
**Total (95% CI)**			**5708**			**5234**	**100.0%**	**14.39 [4.21, 24.58]**
Heterogeneity: Tau² = 402.21; Chi² = 347.79, df = 30 (P < 0.00001); I² = 91%								
Test for overall effect: Z = 2.77 (P = 0.006)								
Test for subgroup differences: Chi² = 2.16, df = 4 (P = 0.71), I² = 0%								

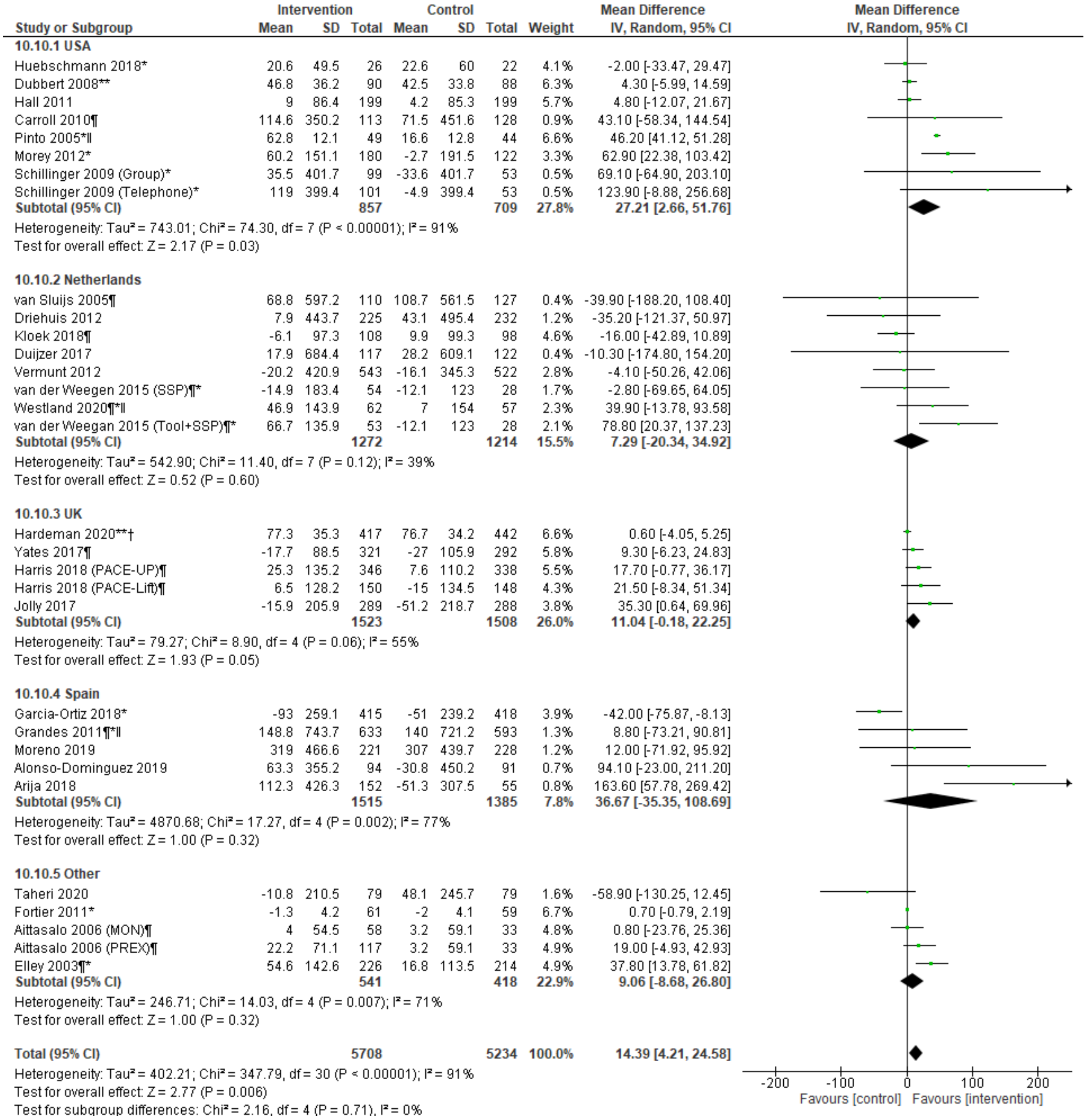

*Intention to treat analysis ¶Cluster randomised controlled trial **Follow-up values †Geometric means ‖Adjusted means

Other countries included: Qatar (Taheri 2020), Canada (Fortier 2011), Finland (Aittasalo), New Zealand (Elley 2003).

Supplementary Figure 2: MVPA minutes/week by follow-up length

Study or Subgroup	Intervention Mean	Intervention SD	Intervention Total	Control Mean	Control SD	Control Total	Weight	Mean Difference IV, Random, 95% CI
**10.11.1 0-6 months**								
Huebschmann 2018*	20.6	49.5	26	22.6	60	22	4.1%	-2.00 [-33.47, 29.47]
Hardeman 2020**†	77.3	35.3	417	76.7	34.2	442	6.6%	0.60 [-4.05, 5.25]
Fortier 2011*	-1.3	4.2	61	-2	4.1	59	6.7%	0.70 [-0.79, 2.19]
Aittasalo 2006 (MON)¶	4	54.5	58	3.2	59.1	33	4.8%	0.80 [-23.76, 25.36]
Aittasalo 2006 (PREX)¶	22.2	71.1	117	3.2	59.1	33	4.9%	19.00 [-4.93, 42.93]
Westland 2020¶*‖	46.9	143.9	62	7	154	57	2.3%	39.90 [-13.78, 93.58]
Carroll 2010¶	114.6	350.2	113	71.5	451.6	128	0.9%	43.10 [-58.34, 144.54]
Pinto 2005*‖	62.8	12.1	49	16.6	12.8	44	6.6%	46.20 [41.12, 51.28]
**Subtotal (95% CI)**			**903**			**818**	**37.0%**	**14.54 [-3.26, 32.35]**
Heterogeneity: Tau² = 466.50; Chi² = 290.36, df = 7 (P < 0.00001); I² = 98%								
Test for overall effect: Z = 1.60 (P = 0.11)								
**10.11.2 7-12 months**								
Taheri 2020	-10.8	210.5	79	48.1	245.7	79	1.6%	-58.90 [-130.25, 12.45]
Garcia-Ortiz 2018*	-93	259.1	415	-51	239.2	418	3.9%	-42.00 [-75.87, -8.13]
van Sluijs 2005¶	68.8	597.2	110	108.7	561.5	127	0.4%	-39.90 [-188.20, 108.40]
Kloek 2018¶	-6.1	97.3	108	9.9	99.3	98	4.6%	-16.00 [-42.89, 10.89]
van der Weegen 2015 (SSP)¶*	-14.9	183.4	54	-12.1	123	28	1.7%	-2.80 [-69.65, 64.05]
Dubbert 2008**	46.8	36.2	90	42.5	33.8	88	6.3%	4.30 [-5.99, 14.59]
Jolly 2017	-15.9	205.9	289	-51.2	218.7	288	3.8%	35.30 [0.64, 69.96]
Elley 2003¶*	54.6	142.6	226	16.8	113.5	214	4.9%	37.80 [13.78, 61.82]
Morey 2012*	60.2	151.1	180	-2.7	191.5	122	3.3%	62.90 [22.38, 103.42]
Schillinger 2009 (Group)*	35.5	401.7	99	-33.6	401.7	53	0.5%	69.10 [-64.90, 203.10]
van der Weegan 2015 (Tool+SSP)¶*	66.7	135.9	53	-12.1	123	28	2.1%	78.80 [20.37, 137.23]
Alonso-Dominguez 2019	63.3	355.2	94	-30.8	450.2	91	0.7%	94.10 [-23.00, 211.20]
Schillinger 2009 (Telephone)*	119	399.4	101	-4.9	399.4	53	0.5%	123.90 [-8.88, 256.68]
Arija 2018	112.3	426.3	152	-51.3	307.5	55	0.8%	163.60 [57.78, 269.42]
**Subtotal (95% CI)**			**2050**			**1742**	**35.0%**	**22.95 [0.08, 45.82]**
Heterogeneity: Tau² = 994.78; Chi² = 50.70, df = 13 (P < 0.00001); I² = 74%								
Test for overall effect: Z = 1.97 (P = 0.05)								
**10.11.3 >12 months**								
Driehuis 2012	7.9	443.7	225	43.1	495.4	232	1.2%	-35.20 [-121.37, 50.97]
Duijzer 2017	17.9	684.4	117	28.2	609.1	122	0.4%	-10.30 [-174.80, 154.20]
Vermunt 2012	-20.2	420.9	543	-16.1	345.3	522	2.8%	-4.10 [-50.26, 42.06]
Hall 2011	9	86.4	199	4.2	85.3	199	5.7%	4.80 [-12.07, 21.67]
Grandes 2011¶*‖	148.8	743.7	633	140	721.2	593	1.3%	8.80 [-73.21, 90.81]
Yates 2017¶	-17.7	88.5	321	-27	105.9	292	5.8%	9.30 [-6.23, 24.83]
Moreno 2019	319	466.6	221	307	439.7	228	1.2%	12.00 [-71.92, 95.92]
Harris 2018 (PACE-UP)¶	25.3	135.2	346	7.6	110.2	338	5.5%	17.70 [-0.77, 36.17]
Harris 2018 (PACE-Lift)¶	6.5	128.2	150	-15	134.5	148	4.3%	21.50 [-8.34, 51.34]
**Subtotal (95% CI)**			**2755**			**2674**	**28.0%**	**10.08 [1.18, 18.97]**
Heterogeneity: Tau² = 0.00; Chi² = 3.09, df = 8 (P = 0.93); I² = 0%								
Test for overall effect: Z = 2.22 (P = 0.03)								
**Total (95% CI)**			**5708**			**5234**	**100.0%**	**14.39 [4.21, 24.58]**
Heterogeneity: Tau² = 402.21; Chi² = 347.79, df = 30 (P < 0.00001); I² = 91%								
Test for overall effect: Z = 2.77 (P = 0.006)								
Test for subgroup differences: Chi² = 1.13, df = 2 (P = 0.57), I² = 0%								

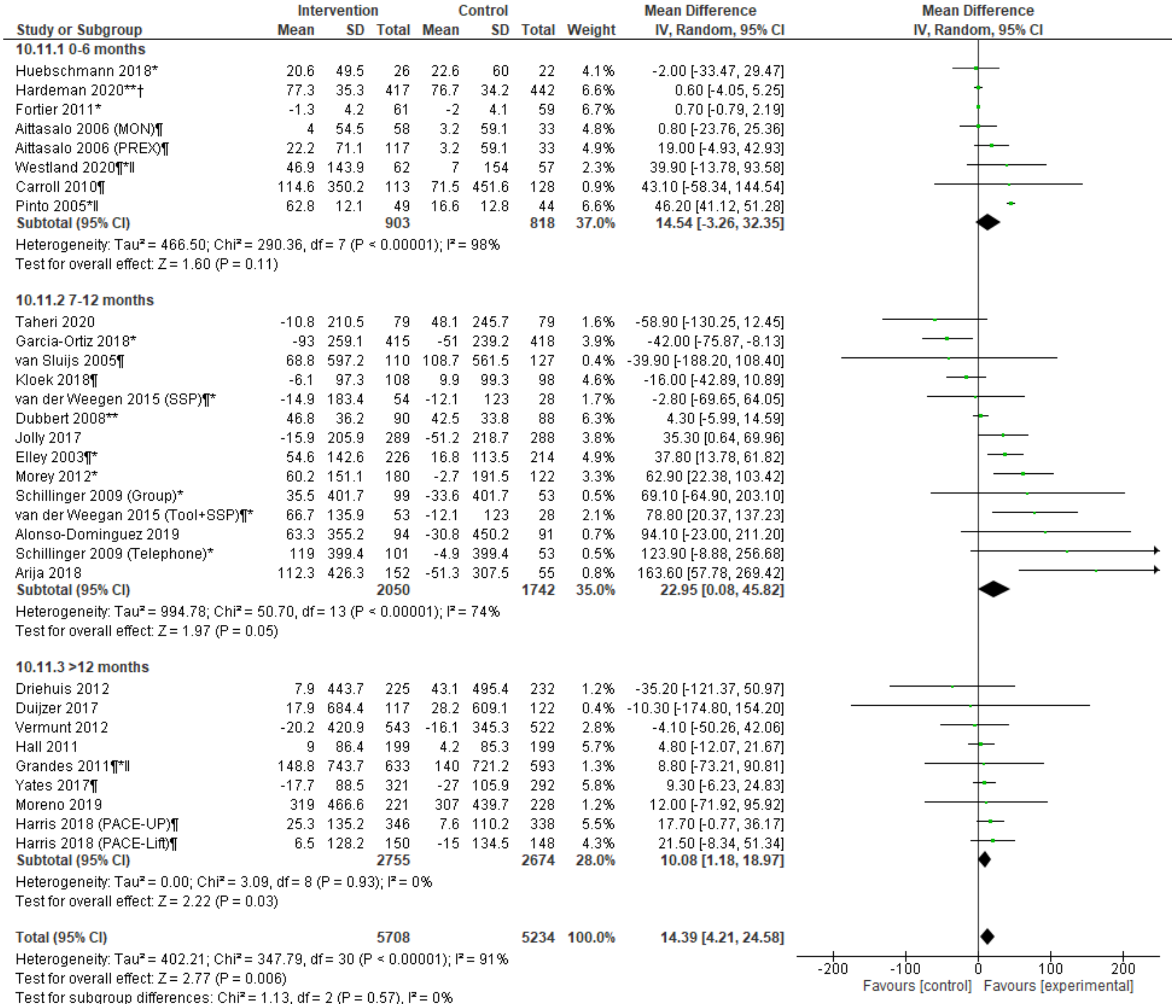

*Intention to treat analysis ¶Cluster randomised controlled trial **Follow-up values †Geometric means ‖Adjusted means

Supplementary Figure 3: Proportion of participants meeting MVPA guidelines according to the number of contacts participants received with an intervenor

Study or Subgroup	Intervention Events	Intervention Total	Control Events	Control Total	Weight	Odds Ratio IV, Random, 95% CI
**1.5.1 Brief**						
Pinto 2002	29	112	35	131	3.5%	0.96 [0.54, 1.70]
Harari 2008¶	6	57	5	64	0.9%	1.39 [0.40, 4.82]
Apinaniz 2019	21	28	14	25	1.0%	2.36 [0.74, 7.55]
**Subtotal (95% CI)**		**197**		**220**	**5.5%**	**1.18 [0.73, 1.89]**
Total events	56		54			
Heterogeneity: Tau² = 0.00; Chi² = 1.93, df = 2 (P = 0.38); I² = 0%						
Test for overall effect: Z = 0.67 (P = 0.51)						
**1.5.2 Multiple brief**						
van Sluijs 2005¶	54	110	73	127	4.2%	0.71 [0.43, 1.19]
Valve 2013¶	43	126	95	264	5.1%	0.92 [0.59, 1.44]
Gomez-Huelgas 2015	50	230	34	176	4.5%	1.16 [0.71, 1.89]
Goldstein 1999¶	35	126	30	125	3.6%	1.22 [0.69, 2.15]
Hesselink 2013¶	83	107	64	87	2.8%	1.24 [0.64, 2.40]
Migneault 2012*	62	169	52	168	5.0%	1.29 [0.82, 2.03]
Lawton 2008*	214	544	179	545	9.4%	1.33 [1.03, 1.70]
Elley 2003¶*	73	226	56	214	5.6%	1.35 [0.89, 2.03]
Morey 2012*	76	180	38	122	4.5%	1.62 [1.00, 2.62]
Tiessen 2013	75	89	65	90	2.4%	2.06 [0.99, 4.29]
Dubbert 2008	58	90	40	88	3.3%	2.17 [1.19, 3.97]
Morey 2009	57	178	28	177	4.2%	2.51 [1.50, 4.18]
Dutton 2006¶	34	38	35	46	0.9%	2.67 [0.77, 9.21]
Clapperton 2020	49	63	31	61	2.1%	3.39 [1.56, 7.37]
**Subtotal (95% CI)**		**2276**		**2290**	**57.5%**	**1.43 [1.18, 1.73]**
Total events	963		820			
Heterogeneity: Tau² = 0.06; Chi² = 25.65, df = 13 (P = 0.02); I² = 49%						
Test for overall effect: Z = 3.60 (P = 0.0003)						
**1.5.3 Intensive**						
Hellgren 2020	8	40	10	26	1.1%	0.40 [0.13, 1.21]
Mitchell 2013	4	26	5	29	0.7%	0.87 [0.21, 3.67]
Driehuis 2012	82	111	101	137	3.6%	1.01 [0.57, 1.78]
Jimmy 2005	26	55	36	77	2.6%	1.02 [0.51, 2.04]
Cheng 2018*	135	170	125	163	4.1%	1.17 [0.70, 1.97]
Moreno 2019	135	221	129	228	6.3%	1.20 [0.83, 1.76]
Harris 2012¶	142	216	108	182	5.7%	1.31 [0.87, 1.98]
Schillinger 2009 (Group)*	60	99	28	53	2.7%	1.37 [0.70, 2.69]
The Activity Counseling Trial 2001 (Assistance)	58	275	21	133	3.8%	1.43 [0.82, 2.47]
The Activity Counseling Trial 2001 (Counseling)	56	260	21	133	3.7%	1.46 [0.84, 2.54]
Schillinger 2009 (Telephone)*	69	101	28	53	2.7%	1.93 [0.97, 3.81]
**Subtotal (95% CI)**		**1574**		**1214**	**37.0%**	**1.24 [1.04, 1.47]**
Total events	775		612			
Heterogeneity: Tau² = 0.00; Chi² = 7.48, df = 10 (P = 0.68); I² = 0%						
Test for overall effect: Z = 2.46 (P = 0.01)						
**Total (95% CI)**		**4047**		**3724**	**100.0%**	**1.33 [1.17, 1.50]**
Total events	1794		1486			
Heterogeneity: Tau² = 0.03; Chi² = 36.22, df = 27 (P = 0.11); I² = 25%						
Test for overall effect: Z = 4.51 (P < 0.00001)						
Test for subgroup differences: Chi² = 1.37, df = 2 (P = 0.50), I² = 0%						

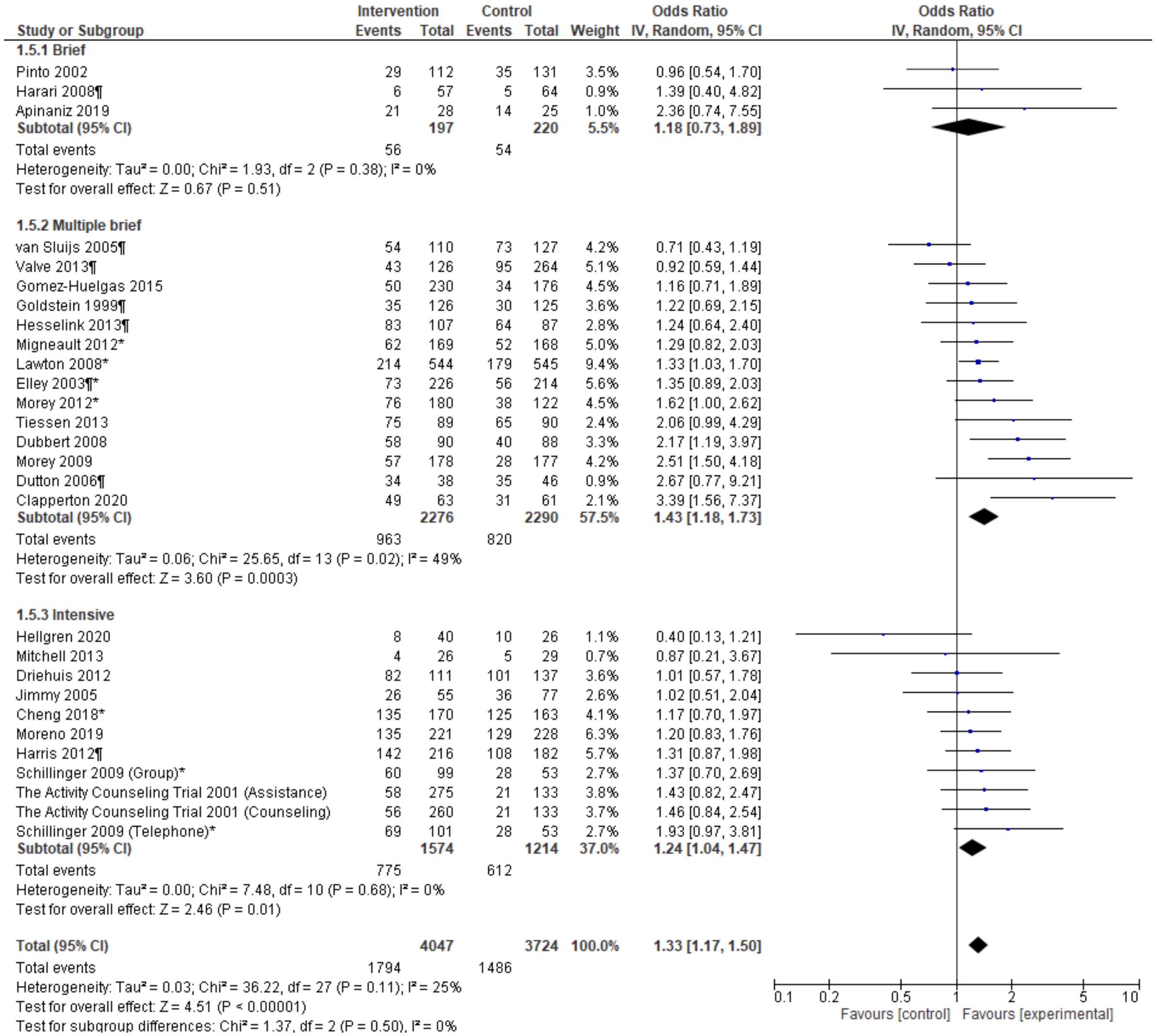

*Intention to treat analysis ¶Cluster randomised controlled trial

Supplementary Figure 4: Total physical activity (i.e., all intensities of physical activity)

Study or Subgroup	Experimental Mean	Experimental SD	Experimental Total	Control Mean	Control SD	Control Total	Weight	Std. Mean Difference IV, Random, 95% CI
**5.1.2 Device-measured**								
Garcia-Ortiz 2018*	-21	82.9	415	-10	72.8	418	4.6%	-0.14 [-0.28, -0.00]
Pears 2016 (Combined)**	632	150.9	52	636	117	37	3.6%	-0.03 [-0.45, 0.39]
Yates 2017¶	-9.3	444.7	228	-39.5	93.3	225	4.5%	0.09 [-0.09, 0.28]
Huebschmann 2018*	3,154.5	13,179.5	26	1,580.6	15,255.5	22	3.1%	0.11 [-0.46, 0.68]
Pears 2016 (Motivational)**	656	205.2	54	636	117	37	3.7%	0.11 [-0.31, 0.53]
Pears 2016 (Pedometer)**	659	233.9	37	636	117	37	3.5%	0.12 [-0.33, 0.58]
Harris 2018 (PACE-Lift)¶	-17	2,696.2	107	-357	2,703.2	116	4.3%	0.13 [-0.14, 0.39]
Fortier 2011*	-13.7	70.1	61	-22.9	60.9	59	3.9%	0.14 [-0.22, 0.50]
Harris 2018 (PACE-UP)¶	478	2,837.3	230	-98	2,422.7	213	4.5%	0.22 [0.03, 0.40]
Mitchell 2013	68.5	356.7	39	-19	336.6	44	3.6%	0.25 [-0.18, 0.68]
Gomez-Huelgas 2015	42.9	35.8	99	-10	9.1	101	4.0%	2.03 [1.68, 2.37]
Pinto 2005*‖	36.3	15	49	-22.7	15.5	44	2.6%	3.84 [3.14, 4.54]
**Subtotal (95% CI)**			**1397**			**1353**	**45.8%**	**0.53 [0.14, 0.92]**
Heterogeneity: Tau² = 0.43; Chi² = 242.07, df = 11 (P < 0.00001); I² = 95%								
Test for overall effect: Z = 2.66 (P = 0.008)								
**5.1.3 Self-reported**								
Driehuis 2012	-167	1,321	111	-92	1,218	137	4.3%	-0.06 [-0.31, 0.19]
Duijzer 2017	159.2	1,552.6	117	98.8	1,094.6	122	4.3%	0.04 [-0.21, 0.30]
Aittasalo 2006 (MON)¶	117	588.7	47	58	691.7	27	3.4%	0.09 [-0.38, 0.57]
Lawton 2008*‡	75	146.1	544	60	136.2	545	4.6%	0.11 [-0.01, 0.23]
Jolly 2017	-27.9	4,037.9	191	-527.7	3,957.7	223	4.5%	0.12 [-0.07, 0.32]
Dubbert 2008	606	3,221.6	99	62	2,465.3	101	4.2%	0.19 [-0.09, 0.47]
Pinto 2002	0.6	2.8	112	0.1	2.4	126	4.3%	0.19 [-0.06, 0.45]
Taheri 2020	190.5	2,272.1	47	-202.9	1,560.3	57	3.8%	0.20 [-0.18, 0.59]
Elley 2003¶*	9.76	42.3	226	0.4	39.5	214	4.5%	0.23 [0.04, 0.42]
Vermunt 2012	-192	919	296	-357	4.7	259	4.5%	0.25 [0.08, 0.41]
Aittasalo 2006 (PREX)¶	204	461.9	89	58	691.7	27	3.6%	0.28 [-0.15, 0.71]
Alonso-Dominguez 2019	438	2,104.3	94	-627	2,953	91	4.2%	0.41 [0.12, 0.71]
Arija 2018	1,054.7	2,704.8	152	-101.8	2,438.8	55	4.1%	0.44 [0.13, 0.75]
**Subtotal (95% CI)**			**2125**			**1984**	**54.2%**	**0.17 [0.11, 0.24]**
Heterogeneity: Tau² = 0.00; Chi² = 12.57, df = 12 (P = 0.40); I² = 4%								
Test for overall effect: Z = 5.18 (P < 0.00001)								
**Total (95% CI)**			**3522**			**3337**	**100.0%**	**0.32 [0.15, 0.49]**
Heterogeneity: Tau² = 0.15; Chi² = 254.81, df = 24 (P < 0.00001); I² = 91%								
Test for overall effect: Z = 3.79 (P = 0.0002)								
Test for subgroup differences: Chi² = 3.14, df = 1 (P = 0.08), I² = 68.2%								

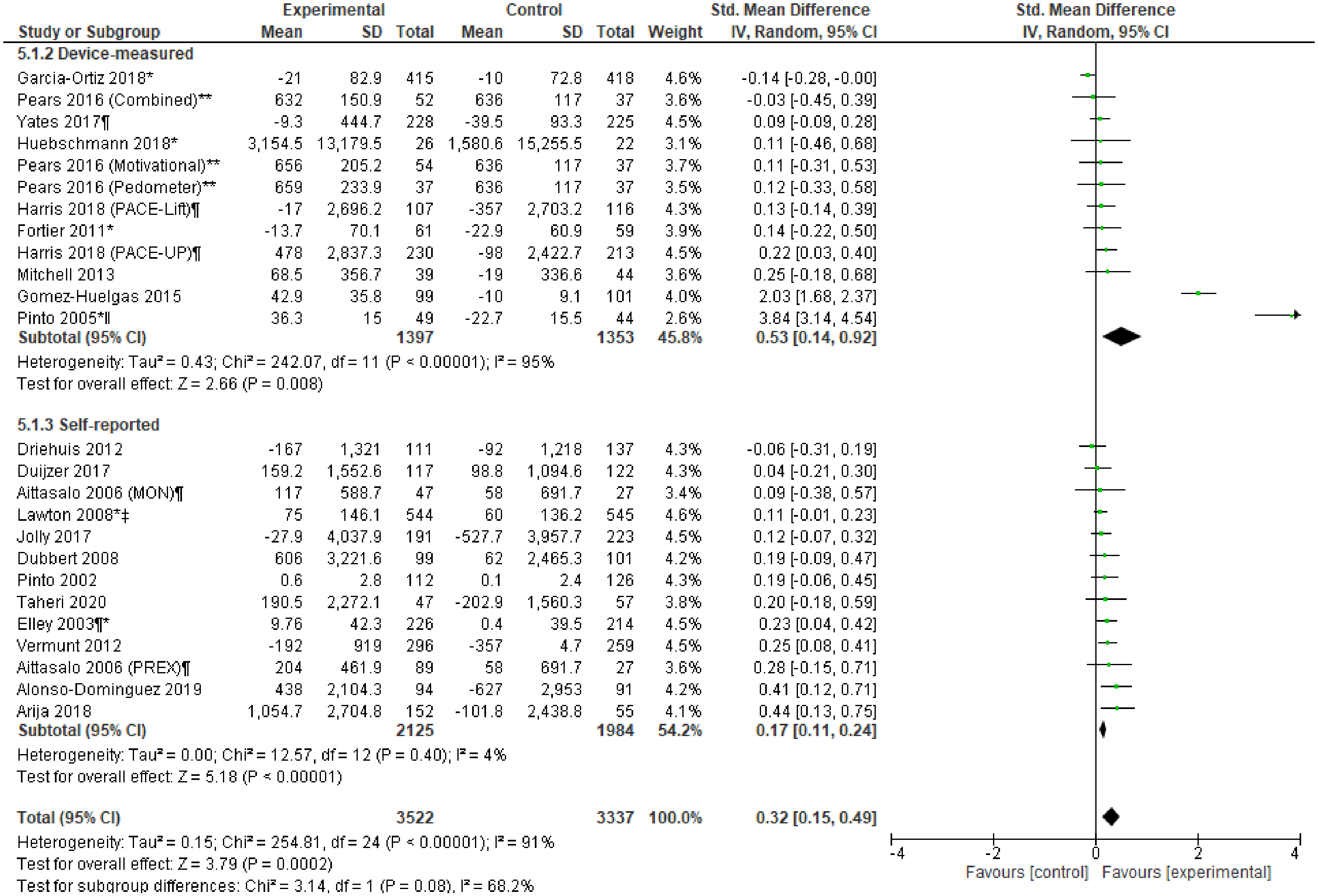

*Intention to treat analysis ¶Cluster randomised controlled trial ‡Medians **Follow-up values ‖Adjusted means

Supplementary Figure 5: Sedentary time minutes per day

Study or Subgroup	Experimental Mean	Experimental SD	Experimental Total	Control Mean	Control SD	Control Total	Weight	Mean Difference IV, Random, 95% CI
Taheri 2020	-40.8	260.3	39	68.8	191	48	0.8%	-109.60 [-207.55, -11.65]
Alonso-Dominguez 2019	-34.2	112.3	94	-7.5	151.7	91	4.0%	-26.70 [-65.26, 11.86]
Pears 2016 (Motivational)**	162.6	82.9	59	182.4	50.7	40	7.0%	-19.80 [-46.15, 6.55]
Pears 2016 (Pedometer)**	163.2	78	43	182.4	50.7	40	6.4%	-19.20 [-47.31, 8.91]
Reed 2008 (counseling)	-47.4	25.8	44	-28.2	28.5	17	12.0%	-19.20 [-34.75, -3.65]
Harris 2018 (PACE-UP)¶	1	66.2	230	2	62.2	213	14.2%	-1.00 [-12.96, 10.96]
Hardeman 2020**†	168	124.8	418	168	127.9	439	11.2%	0.00 [-16.92, 16.92]
Yates 2017¶	23.8	92.3	228	22.9	75.7	225	12.0%	0.90 [-14.64, 16.44]
Pears 2016 (Combined)**	186.6	82.9	59	182.4	50.7	40	7.0%	4.20 [-22.15, 30.55]
Harris 2018 (PACE-Lift)¶	-3	58.1	107	-8	61.4	116	11.9%	5.00 [-10.69, 20.69]
Reed 2008 (counseling & map)	-13.2	28.6	35	-28.2	28.5	17	11.4%	15.00 [-1.53, 31.53]
Kloek 2018¶‖	25.5	173.6	56	-12.7	207.9	50	1.3%	38.20 [-35.20, 111.60]
Garcia-Ortiz 2018*	180	642.5	415	116	603.3	418	1.0%	64.00 [-20.65, 148.65]
**Total (95% CI)**			**1827**			**1754**	**100.0%**	**-3.11 [-11.83, 5.60]**

Heterogeneity: $Tau^2 = 102.31$; $Chi^2 = 22.85$, df = 12 (P = 0.03); $I^2 = 47\%$

Test for overall effect: Z = 0.70 (P = 0.48)

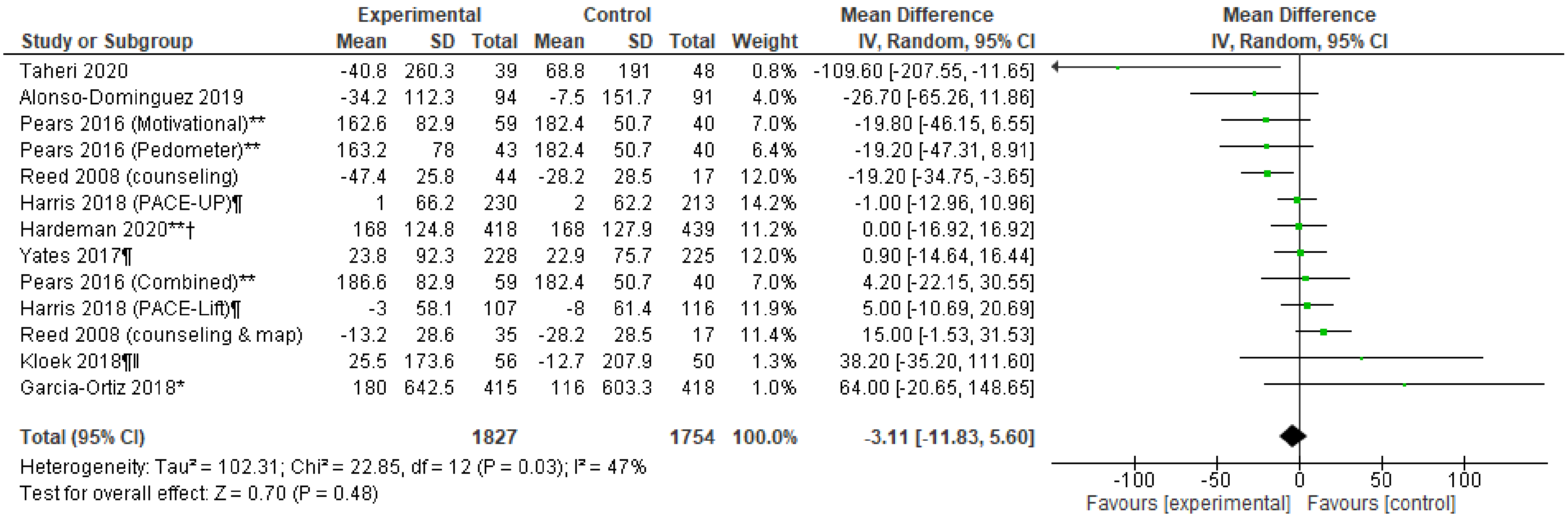

*Intention to treat analysis ¶Cluster randomised controlled trial **Follow-up values †Geometric means ‖Adjusted means

Supplementary Figure 6: Weight (kg)

Study or Subgroup	Experimental Mean	Experimental SD	Experimental Total	Control Mean	Control SD	Control Total	Weight	Mean Difference IV, Random, 95% CI
Apinaniz 2019	-4.3	4.9	33	-5	4	33	3.8%	0.70 [-1.46, 2.86]
Gomez-Huelgas 2015	0.4	9.5	230	0.3	2.4	176	6.0%	0.10 [-1.18, 1.38]
Yates 2017¶	-0.8	5.4	257	-0.9	8	258	6.3%	0.10 [-1.08, 1.28]
van Sluijs 2005¶	-0.9	5	99	-1	3.7	115	6.2%	0.10 [-1.09, 1.29]
Harris 2012¶	-0.1	7.1	216	0.1	8.3	182	5.3%	-0.20 [-1.73, 1.33]
Hesselink 2013¶	-1.4	16	139	-1.1	18.3	101	1.3%	-0.30 [-4.75, 4.15]
Driehuis 2012	-1.4	5.4	148	-1	5.2	165	6.3%	-0.40 [-1.58, 0.78]
Steptoe 1999¶	-0.6	6.6	95	-0.2	5.7	198	5.2%	-0.40 [-1.95, 1.15]
Lawton 2008*	-0.6	4	544	-0.2	3.4	545	8.2%	-0.40 [-0.84, 0.04]
Vermunt 2012	-0.8	5.1	305	-0.4	4.7	259	7.4%	-0.40 [-1.21, 0.41]
Morey 2012*	-1.5	3.8	180	-0.8	3.2	122	7.4%	-0.70 [-1.49, 0.09]
Arija 2018	-1.1	4	152	-0.4	3.7	55	6.3%	-0.70 [-1.87, 0.47]
Fortier 2011*	-0.8	2.3	20	0.2	2.2	15	5.3%	-1.00 [-2.50, 0.50]
Volger 2013 (Brief)*‖	-2.9	8	131	-1.7	8	65	3.4%	-1.20 [-3.58, 1.18]
Dutton 2006¶*	-2	3.2	50	0.2	2.9	50	6.2%	-2.20 [-3.40, -1.00]
Duijzer 2017	-2.9	5.1	118	-0.4	3.7	122	6.4%	-2.50 [-3.63, -1.37]
Volger 2013 (Enhanced)*‖	-4.6	8	129	-1.7	8	65	3.4%	-2.90 [-5.28, -0.52]
Hellgren 2020	-3	6.2	43	0.7	6.2	26	2.5%	-3.70 [-6.72, -0.68]
Taheri 2020*	-12	9.5	70	-4	5.3	77	3.1%	-8.00 [-10.52, -5.48]
**Total (95% CI)**			**2959**			**2629**	**100.0%**	**-1.01 [-1.56, -0.45]**

Heterogeneity: Tau² = 0.94; Chi² = 65.13, df = 18 (P < 0.00001); I² = 72%
Test for overall effect: Z = 3.52 (P = 0.0004)

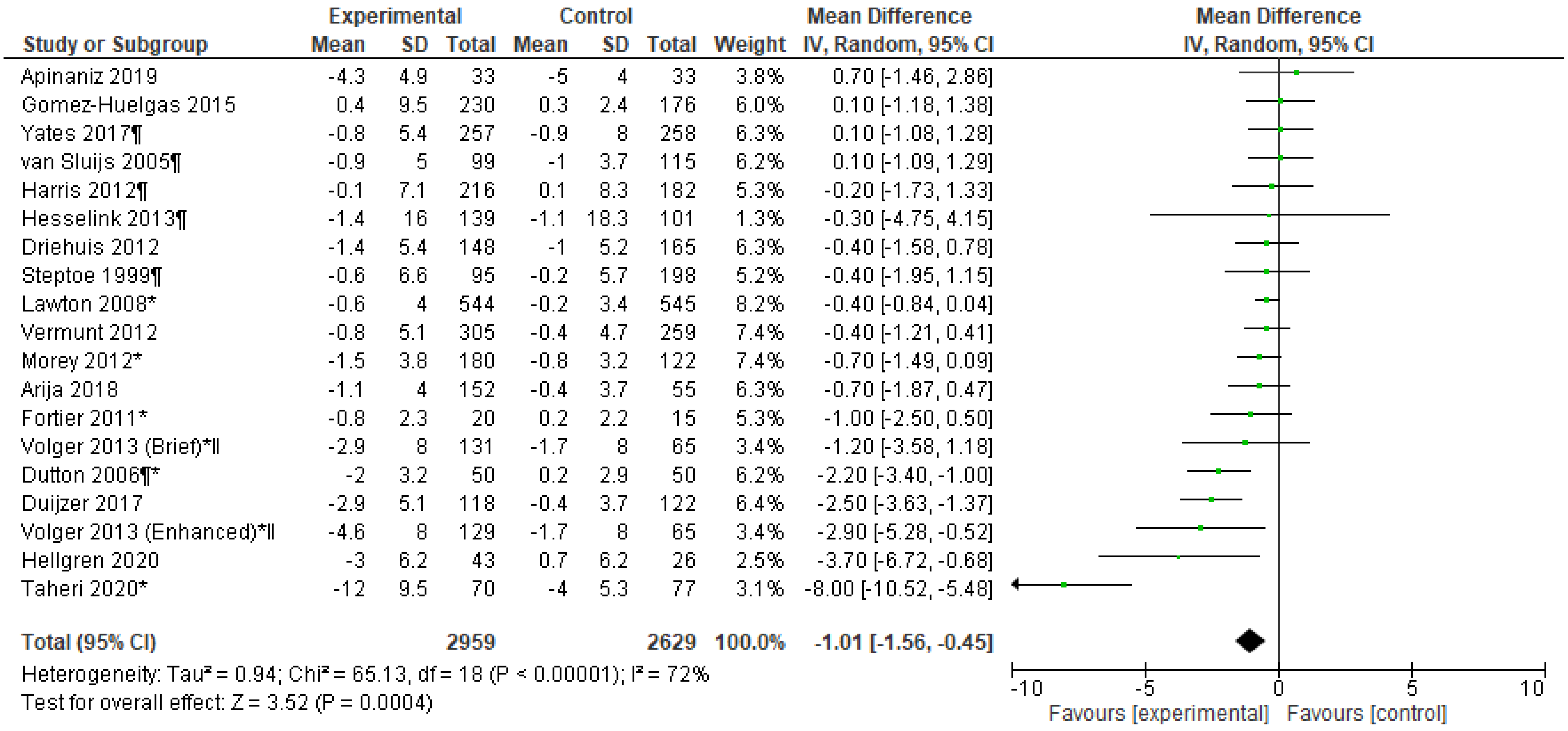

*Intention to treat analysis ¶Cluster randomised controlled trial ‖Adjusted means

Supplementary Figure 7: Body Mass Index (kg/m^2)

Study or Subgroup	Experimental Mean [kg/m2]	Experimental SD [kg/m2]	Experimental Total	Control Mean [kg/m2]	Control SD [kg/m2]	Control Total	Weight	Mean Difference IV, Random, 95% CI
Clapperton 2020	0.4	2.7	63	-0.3	1.4	61	1.7%	0.70 [-0.05, 1.45]
Harris 2012¶	-0.9	1.6	216	-1.3	1.8	182	4.6%	0.40 [0.06, 0.74]
Moreno 2019	-0.1	1.4	248	-0.4	1.2	253	5.9%	0.30 [0.07, 0.53]
Schillinger 2009 (Telephone)*	0.4	1.9	104	0.2	2.1	53	2.0%	0.20 [-0.47, 0.87]
Yates 2017¶	-0.2	2.2	257	-0.4	1.9	258	4.4%	0.20 [-0.16, 0.56]
Harris 2018 (PACE-Lift)¶	-0.1	1.2	146	-0.3	1.1	143	5.5%	0.20 [-0.07, 0.47]
Alonso-Dominguez 2019	-0.3	1	94	-0.4	1	91	5.2%	0.10 [-0.19, 0.39]
Harris 2018 (PACE-UP)¶	-0.1	1.5	321	-0.2	1.3	323	6.1%	0.10 [-0.12, 0.32]
Schillinger 2009 (Group)*	0.3	2.4	104	0.2	2.1	53	1.8%	0.10 [-0.63, 0.83]
van Sluijs 2005¶	-0.2	1.6	99	-0.3	1	115	4.3%	0.10 [-0.26, 0.46]
Duijzer 2017	-1	1.7	118	-1	1.3	122	4.0%	0.00 [-0.38, 0.38]
Elley 2003¶*	-0.1	1.5	226	-0.1	1.4	214	5.4%	0.00 [-0.27, 0.27]
Garcia-Ortiz 2018*	0.1	1.5	415	0.1	1.4	418	6.4%	0.00 [-0.20, 0.20]
Tiessen 2013	-0.1	1.3	89	-0.1	1.5	90	3.8%	0.00 [-0.41, 0.41]
Steptoe 1999¶	-0.2	2.6	95	-0.1	1.9	198	2.4%	-0.10 [-0.69, 0.49]
Gomez-Huelgas 2015	0.1	3.6	230	0.3	2.4	176	2.4%	-0.20 [-0.78, 0.38]
Jimmy 2005	0.1	1.3	55	0.3	1.1	77	3.7%	-0.20 [-0.62, 0.22]
Vermunt 2012	-0.3	1.8	305	-0.1	1.7	259	5.1%	-0.20 [-0.49, 0.09]
Driehuis 2012‖	-0.4	1.8	171	-0.2	1.7	186	4.3%	-0.20 [-0.56, 0.16]
Fortier 2011*	-0.3	0.9	20	0	0.9	15	2.4%	-0.30 [-0.90, 0.30]
Hesselink 2013¶	-0.6	4.9	139	-0.3	5.3	101	0.6%	-0.30 [-1.62, 1.02]
Morey 2012*	-0.6	1.1	180	-0.3	0.9	122	6.0%	-0.30 [-0.53, -0.07]
Arija 2018	-0.5	1.2	152	-0.2	1.1	55	4.4%	-0.30 [-0.65, 0.05]
Volger 2013 (Brief)*‖	-0.9	2.3	131	-0.6	2.3	65	2.0%	-0.30 [-0.98, 0.38]
Kloek 2018¶	-0.9	1.2	66	-0.2	1.2	69	3.8%	-0.70 [-1.10, -0.30]
Volger 2013 (Enhanced)*‖	-1.6	1.8	85	-0.6	2.3	65	2.0%	-1.00 [-1.68, -0.32]
**Total (95% CI)**			**4129**			**3764**	**100.0%**	**-0.04 [-0.15, 0.07]**

Heterogeneity: $Tau^2 = 0.04$; $Chi^2 = 56.34$, df = 25 (P = 0.0003); $I^2 = 56\%$
Test for overall effect: Z = 0.67 (P = 0.50)

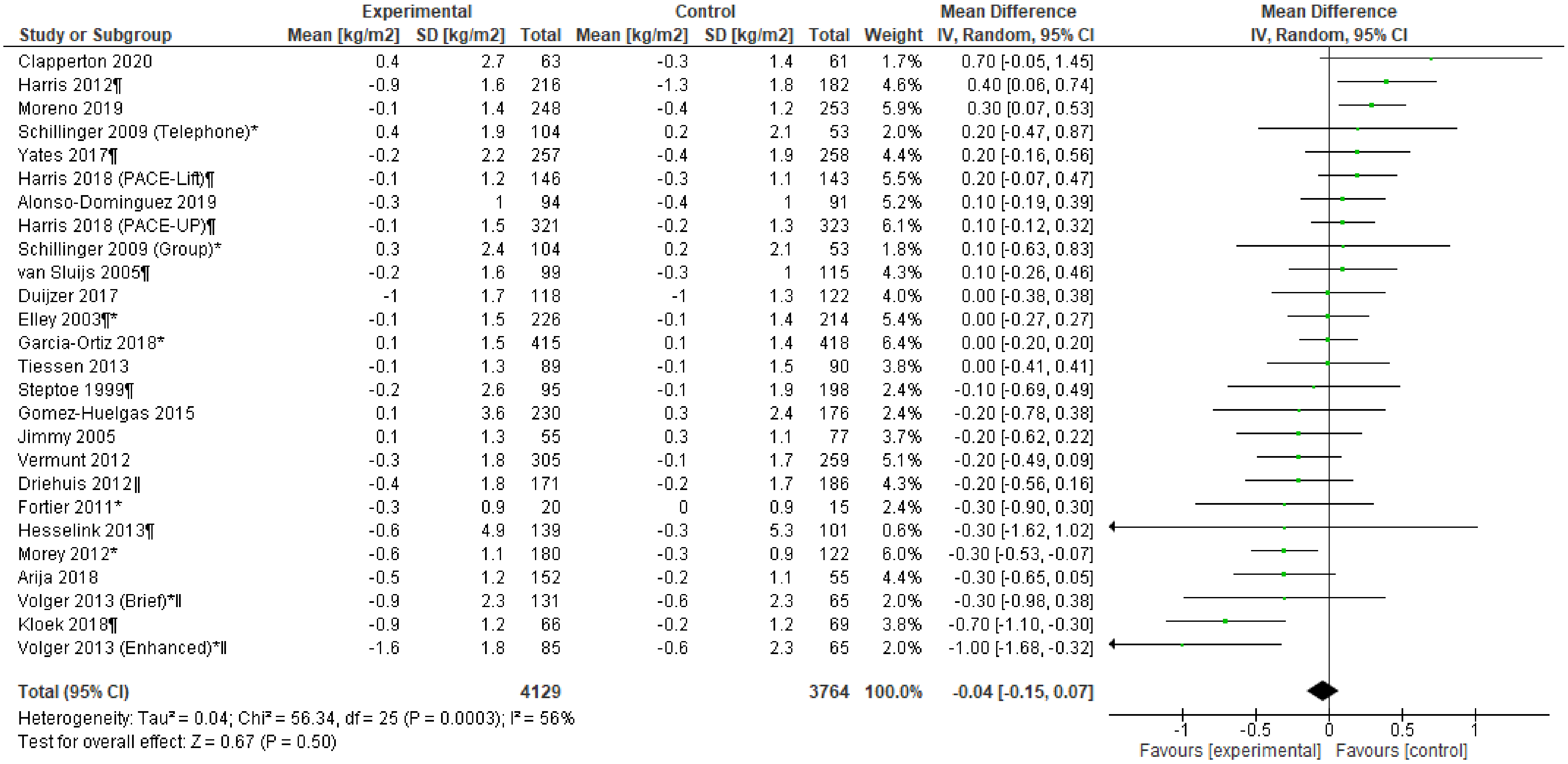

*Intention to treat analysis ¶Cluster randomised controlled trial ‖Adjusted means

Supplementary Figure 8: Risk of bias assessments

year	**Study ID**	**D1**	**D2**	**D3**	**D4**	**D5**	**Overall**
1999	Goldstein 1999	-	+	+	!	!	-
2001	The Activity Counseling Trial 2001	+	+	+	!	+	!
2002	Pinto 2002	!	+	+	!	!	!
2003	Elley 2003	+	+	+	!	!	!
2005	Jimmy 2005	+	+	+	!	!	!
2005	Pinto 2005	!	+	+	!	!	!
2005	van Sluijs 2005	!	+	+	!	!	!
2006	Aittasalo 2006	-	+	+	!	!	-
2006	Dutton 2006	+	+	+	!	!	!
2008	Dubbert 2008	!	+	+	+	!	!
2008	Harari 2008	+	+	+	-	+	-
2008	Lawton 2008	+	+	+	!	+	!
2009	Morey 2009	+	+	+	!	+	!
2009	Schillinger 2009	!	+	+	-	!	-
2010	Carroll 2010	!	+	+	!	!	!
2011	Fortier 2011	+	+	+	+	+	+
2011	Grandes 2011	-	+	+	!	+	-
2012	Driehuis 2012	+	+	+	!	+	!
2012	Harris 2012	+	+	+	!	+	!
2012	Migneault 2012	!	+	+	!	!	!
2012	Morey 2012	!	+	+	!	!	!
2012	Vermunt 2012	-	+	-	!	!	-
2013	Hesselink 2013	+	+	+	!	+	!
2013	Mitchell 2013	+	+	-	+	-	-

+ Low risk

! Some concerns

\- High risk

D1 Randomisation process

D2 Deviations from the intended interventions

D3 Missing outcome data

D4 Measurement of the outcome

D5 Selection of the reported result

2013	Tiessen 2013	+	+	+	!	!	!
2013	Valve 2013	!	+	+	!	!	!
2015	Gomez-Huelgas 2015	!	!	-	!	!	-
2015	van der Weegen 2015	+	+	+	+	+	+
2017	Duijzer 2017	+	+	+	!	+	!
2017	Jolly 2017	+	+	!	+	+	!
2017	Yates 2017	+	+	-	+	+	-
2018	Arija 2018	+	+	+	!	!	!
2018	Cheng 2018	+	+	+	-	+	-
2018	Garcia-Ortiz 2018	+	+	+	+	+	+
2018	Harris 2018 (PACE-Lift)	+	+	+	+	!	!
2018	Harris 2018 (PACE-UP)	+	+	-	+	!	-
2018	Huebschmann 2018	+	+	+	+	!	!
2018	Kloek 2018	+	+	+	+	+	+
2019	Alonso-Dominguez 2019	!	+	+	!	+	!
2019	Apinaniz 2019	+	+	-	!	!	-
2019	Moreno 2019	!	+	+	!	+	!
2020	Clapperton 2020	+	+	+	!	!	!
2020	Hardeman 2020	+	+	+	+	+	+
2020	Hellgren 2020	!	+	-	!	!	-
2020	Taheri 2020	+	+	-	!	+	-
2020	Westland 2020	+	+	+	+	+	+

Supplementary Figure 9: Funnel plot for MVPA minutes per week outcome

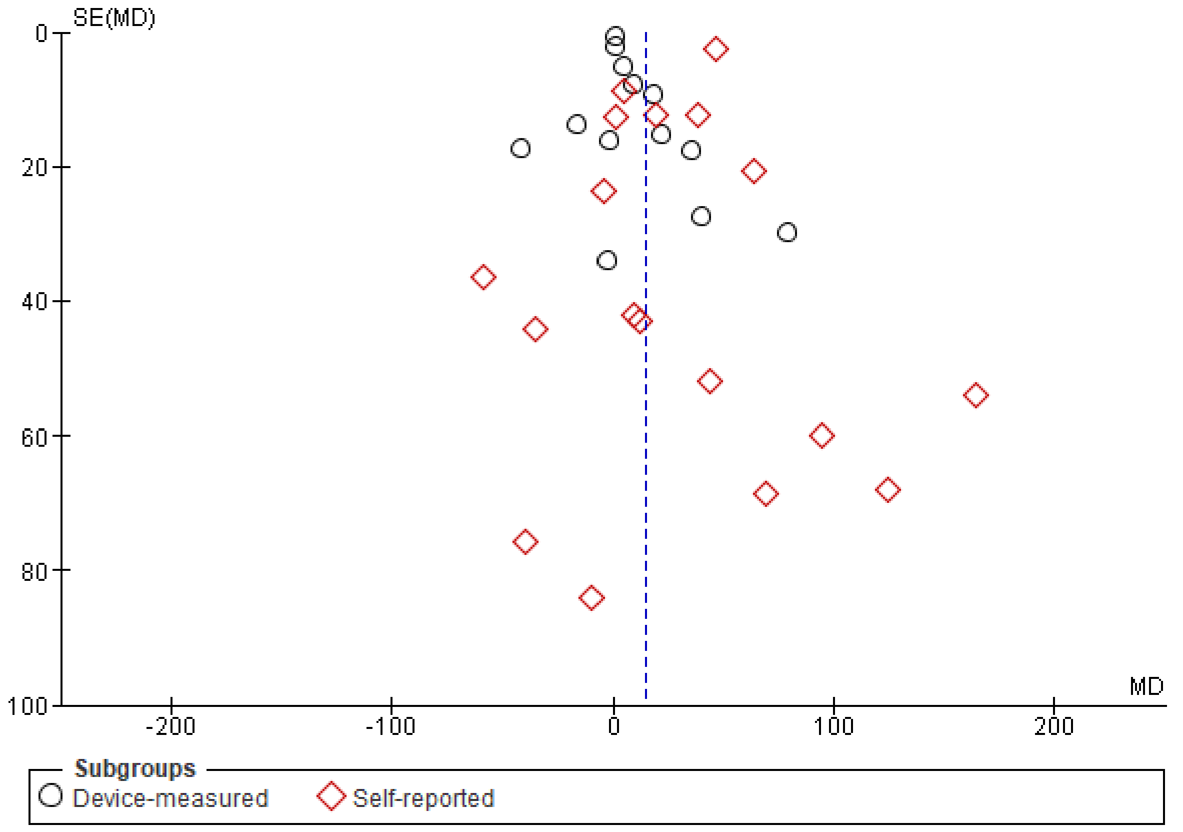

Supplementary Figure 10: Funnel plot for proportion meeting MVPA guidelines outcome

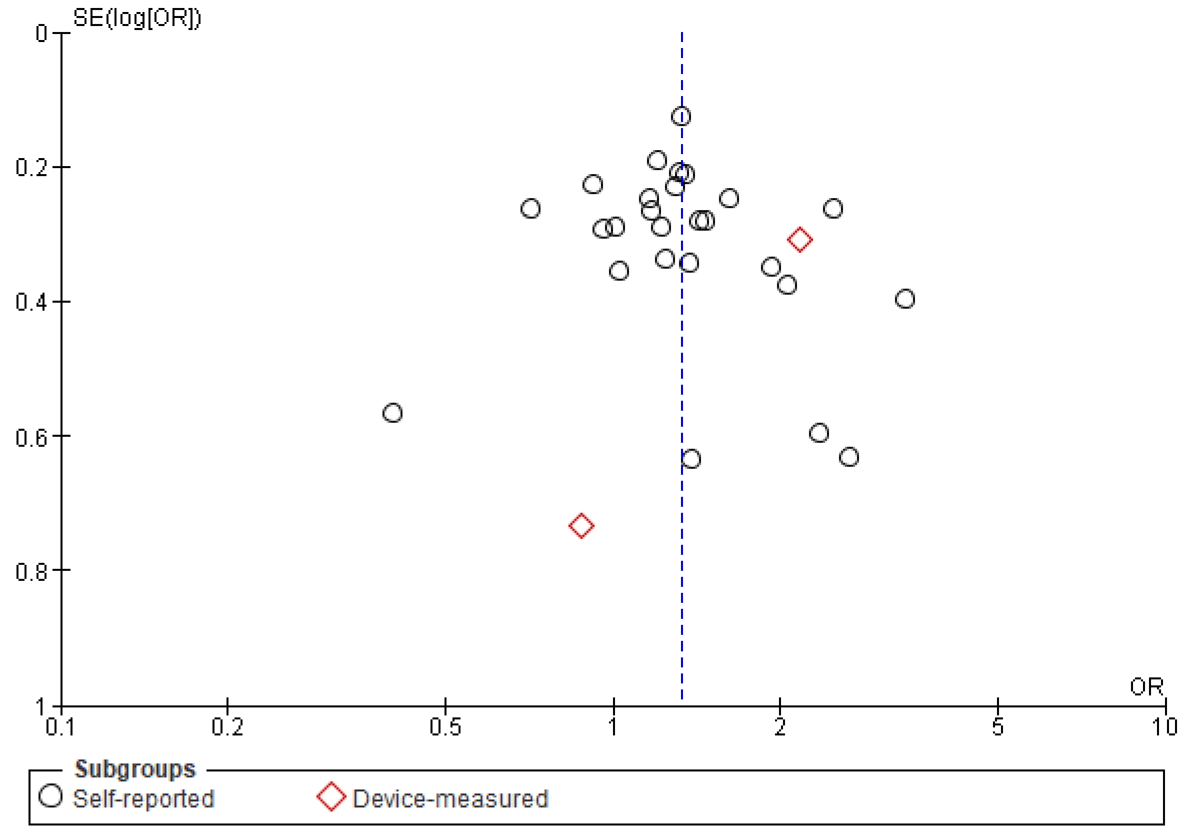

Supplement: Supplementary file 1 — Web appendix: Supplementary materials [file ketv068465.ww.pdf]
